# Supplementary material for: Anti-Pulmonary Fibrosis Activities of Triterpenoids from Oenothera biennis
Source: Molecules. 2022 Jul 29;27(15):4870. doi: 10.3390/molecules27154870 (PMC9369581; doi:10.3390/molecules27154870)
Supplement: Supplementary file 1 [file molecules-27-04870-s001.zip › molecules-1762113-supplementary.pdf]

Supplementary Information

Anti-pulmonary fibrosis activities triterpenoids from the *Oenothera biennis*

Juan-Juan Liu[1,2], Jing-Ke Zhang[1,2], Meng-Nan Zeng[1,2], Meng Li[1,2], Shuang-shuang Xie[1,2], Xiao-ke Zheng[1,2]and Wei-Sheng Feng[1,2*]

1. Henan University of Chinese Medicine, Zhengzhou 450046, China

2. The Engineering and Technology Center for Chinese Medicine Development of Henan Province, Zhengzhou 450046, China

* Correspondence: Wei-Sheng Feng fwsh@hactcm.edu.cn

Abstract:

Five new triterpenoids, oenotheralanosterols C-G (1−5), with seven known triterpenoid compounds, namely 2α,3α,19α-trihydroxy-24-norurs4,12-dien-28-oic acid (6), 3β,23-dihydroxy-1-oxo-olean-12-en-28-oic acid (7), reimagine C (8), knoxivalic acid A (9), termichebulolide (10), rosasecotriterpene A (11), rosanortriterpene C (12), were extracted and separated from the dichloromethane part of Oenothera biennis L. The anti-pulmonary fibrosis activities of all the compounds against TGF-β1 induced damage on normal human lung epithelial (BEAS-2B) cells were investigated in vitro. The results showed that compounds 1−2, 6, 8, and 11 exhibited significant anti-pulmonary fibrosis activities, with EC50 values ranging from 4.7 μM to 9.9 μM.

Keywords: *Oenothera biennis* L.; Triterpenoids; Anti-pulmonary fibrosis activities; BEAS-2B cells; RTCA

Contents

Figure 1. Structures of **1-12**

Figure2. Key ^{1}H-^{1}H COSY and HMBC correlations of compound **1-5**

Figure3. Key NOESY correlations of compound **1-5**

Figure S1. ^{1}H NMR spectrum (500 MHz, CD_3OD) of **1**

Figure S2. ^{13}C NMR spectrum (125 MHz, CD_3OD) of **1**

Figure S3. DEPT 135 spectrum (125 MHz, CD_3OD) of **1**

Figure S4. ^{1}H-^{1}H COSY spectrum of **1**

Figure S5. HSQC spectrum of **1**

Figure S6. HMBC spectrum of **1**

Figure S7. NOESY spectrum of **1**

Figure S8. HR-ESI-MS Spectrum of **1**

Figure S9. UV spectrum of **1**

Figure S10. IR spectrum of **1**

Figure S11. ^{1}H NMR spectrum (500 MHz, CD_3OD) of **2**

Figure S12. ^{13}C NMR spectrum (125 MHz, CD_3OD) of **2**

Figure S13. DEPT 135 spectrum (125 MHz, CD_3OD) of **2**

Figure S14. ^{1}H-^{1}H COSY spectrum of **2**
Figure S15. HSQC spectrum of **2**
Figure S16. HMBC spectrum of **2**
Figure S17. NOESY spectrum of **2**
Figure S18. HR-ESI-MS Spectrum of **2**
Figure S19. UV spectrum of **2**
Figure S20. IR spectrum of **2**
Figure S21. ^{1}H NMR spectrum (500 MHz, CD_3OD) of **3**
Figure S22. ^{13}C NMR spectrum (125 MHz, CD_3OD) of **3**
Figure S23. DEPT 135 spectrum (125 MHz, CD_3OD) of **3**
Figure S24. ^{1}H-^{1}H COSY spectrum of **3**
Figure S25. HSQC spectrum of **3**
Figure S26. HMBC spectrum of **3**
Figure S27. NOESY spectrum of **3**
Figure S28. HR-ESI-MS Spectrum of **3**
Figure S29. UV spectrum of **3**
Figure S30. IR spectrum of **3**
Figure S31. ^{1}H NMR spectrum (500 MHz, CD_3OD) of **4**
Figure S32. ^{13}C NMR spectrum (125 MHz, CD_3OD) of **4**
Figure S33. DEPT 135 spectrum (125 MHz, CD_3OD) of **4**
Figure S34. ^{1}H-^{1}H COSY spectrum of **4**
Figure S35. HSQC spectrum of **4**
Figure S36. HMBC spectrum of **4**
Figure S37. NOESY spectrum of **4**
Figure S38. HR-ESI-MS Spectrum of **4**
Figure S39. UV spectrum of **4**
Figure S40. IR spectrum of **4**
Figure S41. ^{1}H NMR spectrum (500 MHz, CD_3OD) of **5**
Figure S42. ^{13}C NMR spectrum (125 MHz, CD_3OD) of **5**
Figure S43. DEPT 135 spectrum (125 MHz, CD_3OD) of **5**
Figure S44. ^{1}H-^{1}H COSY spectrum of **5**
Figure S45. HSQC spectrum of **5**
Figure S46. HMBC spectrum of **5**
Figure S47. NOESY spectrum of **5**
Figure S48. HR-ESI-MS Spectrum of **5**
Figure S49. UV spectrum of **5**
Figure S50. IR spectrum of **5**
Figure S51. ^{1}H NMR spectrum (500 MHz, CD_3OD) of **6**
Figure S52. ^{13}C NMR spectrum (125 MHz, CD_3OD) of **6**
Figure S53. HR-ESI-MS Spectrum of **6**
Figure S54. ^{1}H NMR spectrum (500 MHz, CD_3OD) of **7**
Figure S55. ^{13}C NMR spectrum (125 MHz, CD_3OD) of **7**
Figure S56. HR-ESI-MS Spectrum of **7**
Figure S57. ^{1}H NMR spectrum (500 MHz, CD_3OD) of **8**

Figure S58. ^{13}C NMR spectrum (125 MHz, CD_3OD) of **8**
Figure S59. HR-ESI-MS Spectrum of **8**
Figure S60. ^{1}H NMR spectrum (500 MHz, Acetone-d_6) of **9**
Figure S61. ^{13}C NMR spectrum (125 MHz, Acetone-d_6) of **9**
Figure S62. HR-ESI-MS Spectrum of **9**
Figure S63. ^{1}H NMR spectrum (500 MHz, CD_3OD) of **10**
Figure S64. ^{13}C NMR spectrum (125 MHz, CD_3OD) of **10**
Figure S65. HR-ESI-MS Spectrum of **10**
Figure S66. ^{1}H NMR spectrum (500 MHz, CD_3OD) of **11**
Figure S67. ^{13}C NMR spectrum (125 MHz, CD_3OD) of **11**
Figure S68. HR-ESI-MS Spectrum of **11**
Figure S69. ^{1}H NMR spectrum (500 MHz, CD_3OD) of **12**
Figure S70. ^{13}C NMR spectrum (125 MHz, CD_3OD) of **12**
Figure S71. HR-ESI-MS Spectrum of **12**
Table 4 Melting points of compounds **6-12**
Figure S72. The curve obtained with compound **1**
Figure S73. The curve obtained with compound **2**
Figure S74. The curve obtained with compound **3**
Figure S75. The curve obtained with compound **6**
Figure S76. The curve obtained with compound **7**
Figure S77. The curve obtained with compound **8**
Figure S78. The curve obtained with compound **11**
Figure S79. The curve obtained with positive control compound Pirfenidone

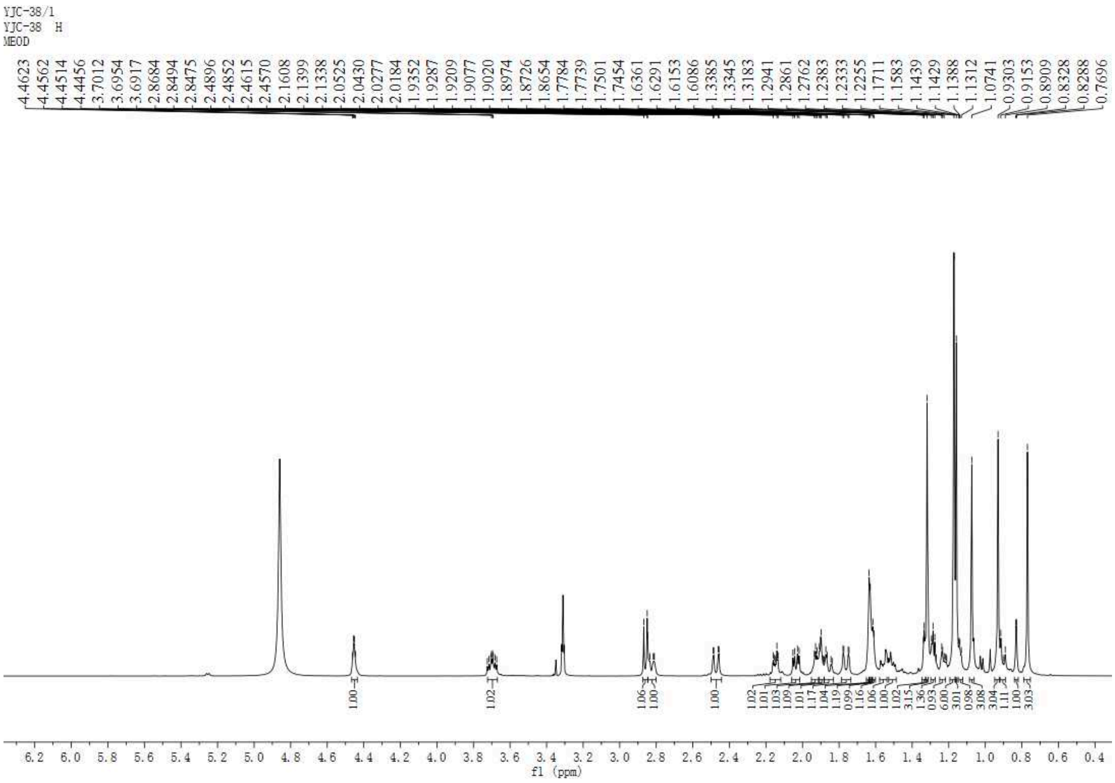

Figure S1. ^{1}H NMR spectrum (500 MHz, CD_3OD) of **1**

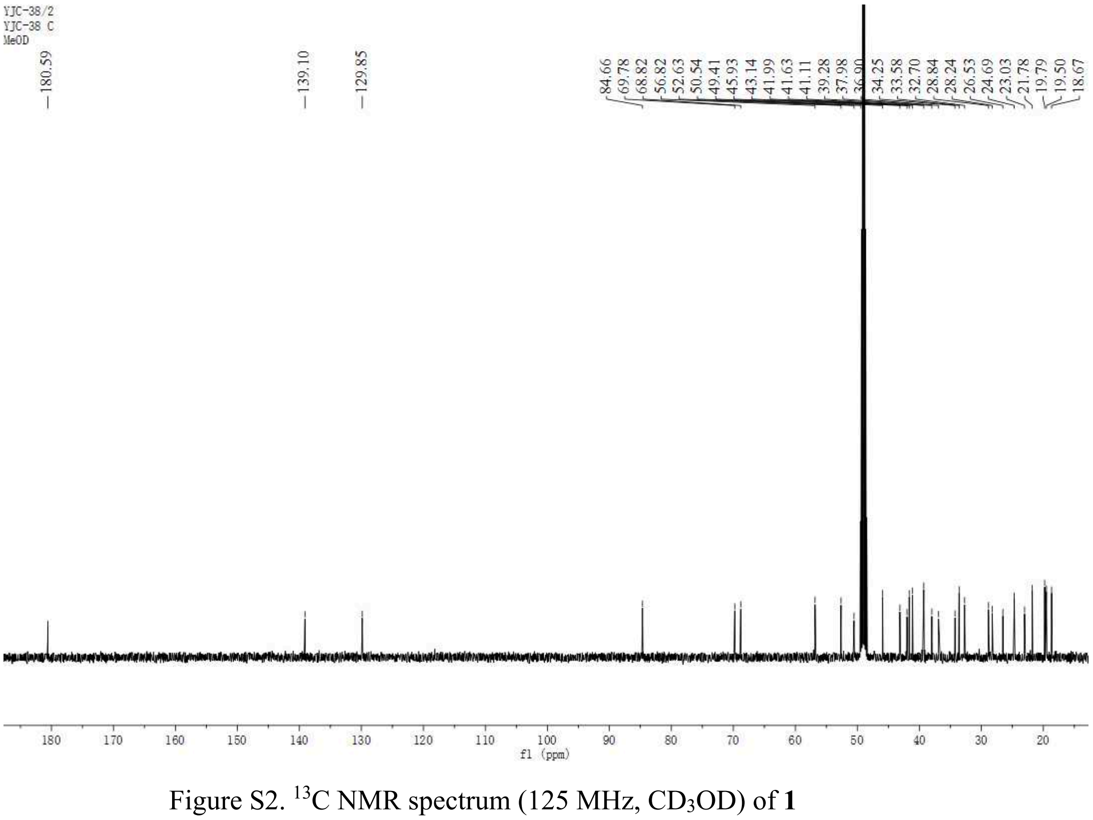

Figure S2. ^{13}C NMR spectrum (125 MHz, CD_3OD) of **1**

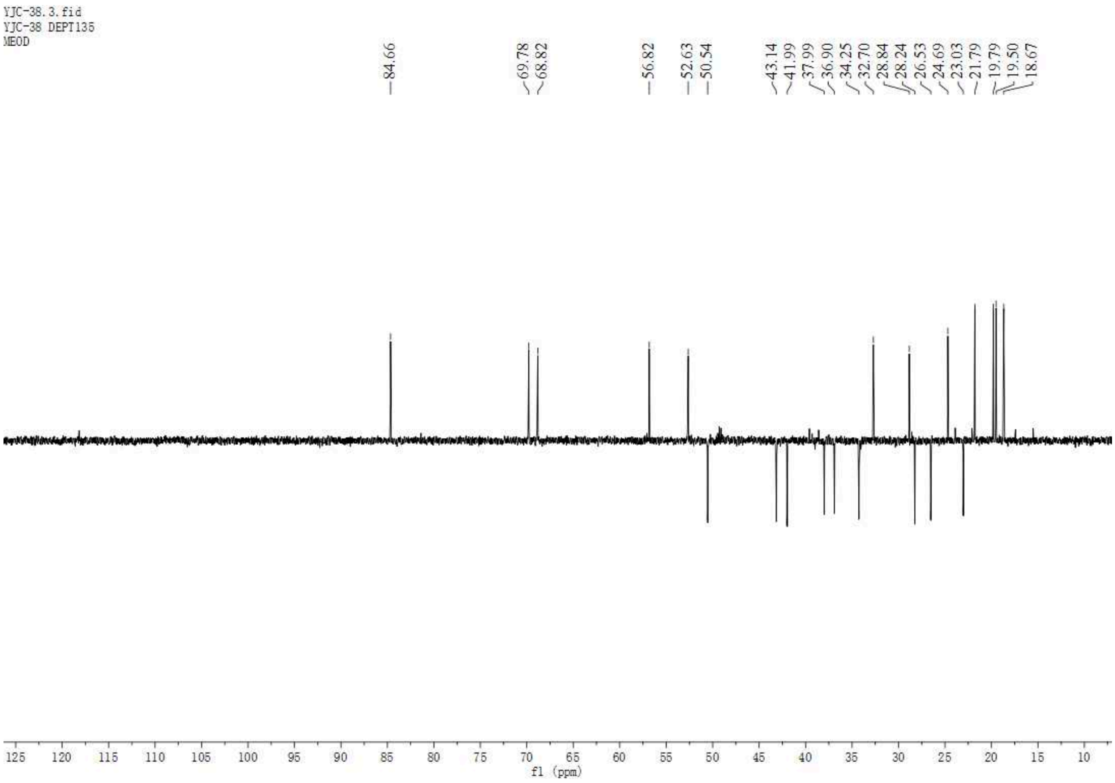

Figure S3. DEPT 135 spectrum (125 MHz, CD_3OD) of **1**

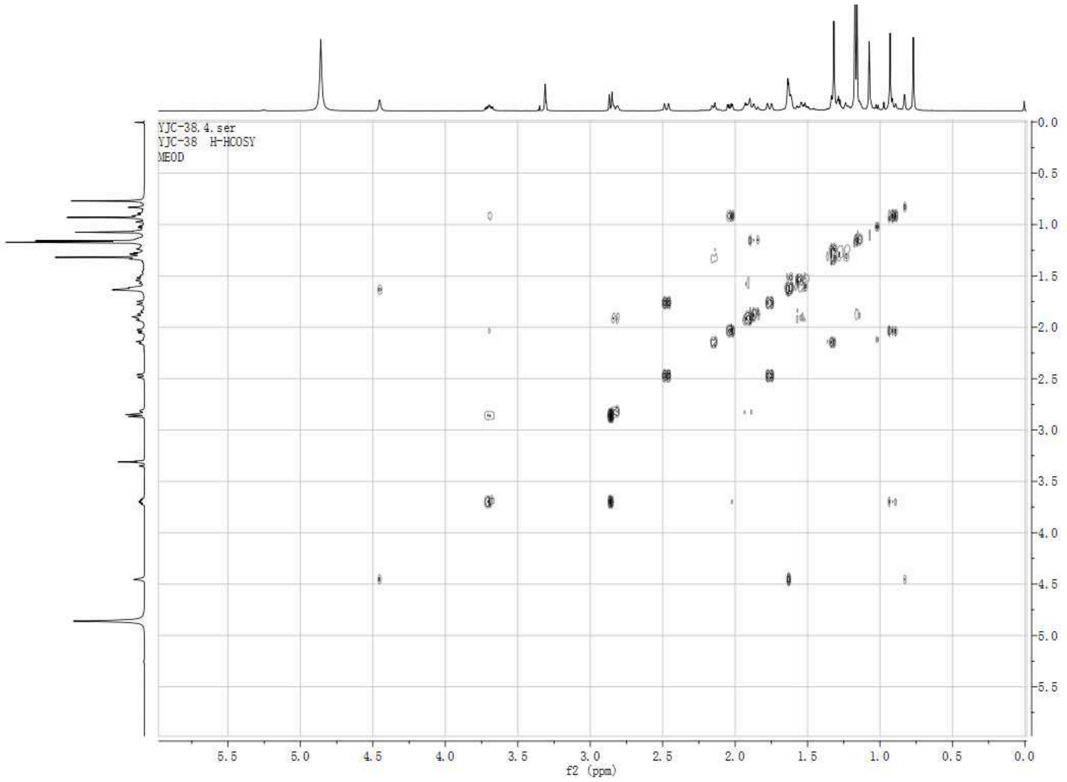

Figure S4. ^{1}H-^{1}H COSY spectrum of **1**

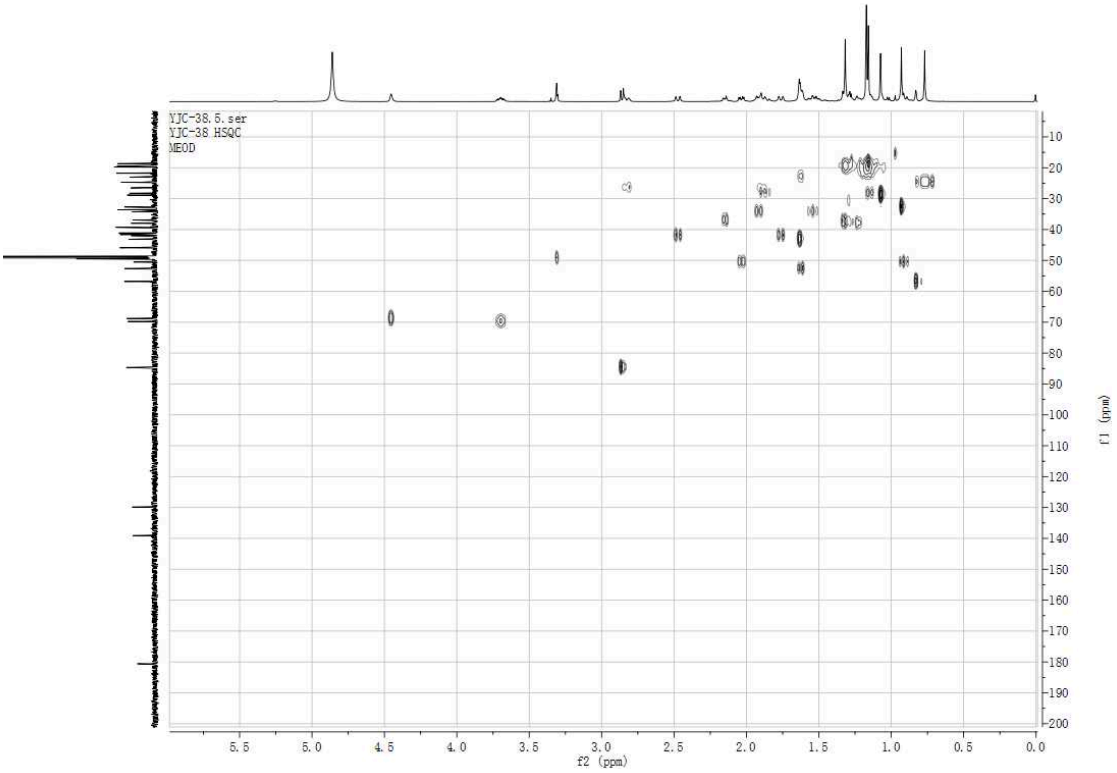

Figure S5. HSQC spectrum of **1**

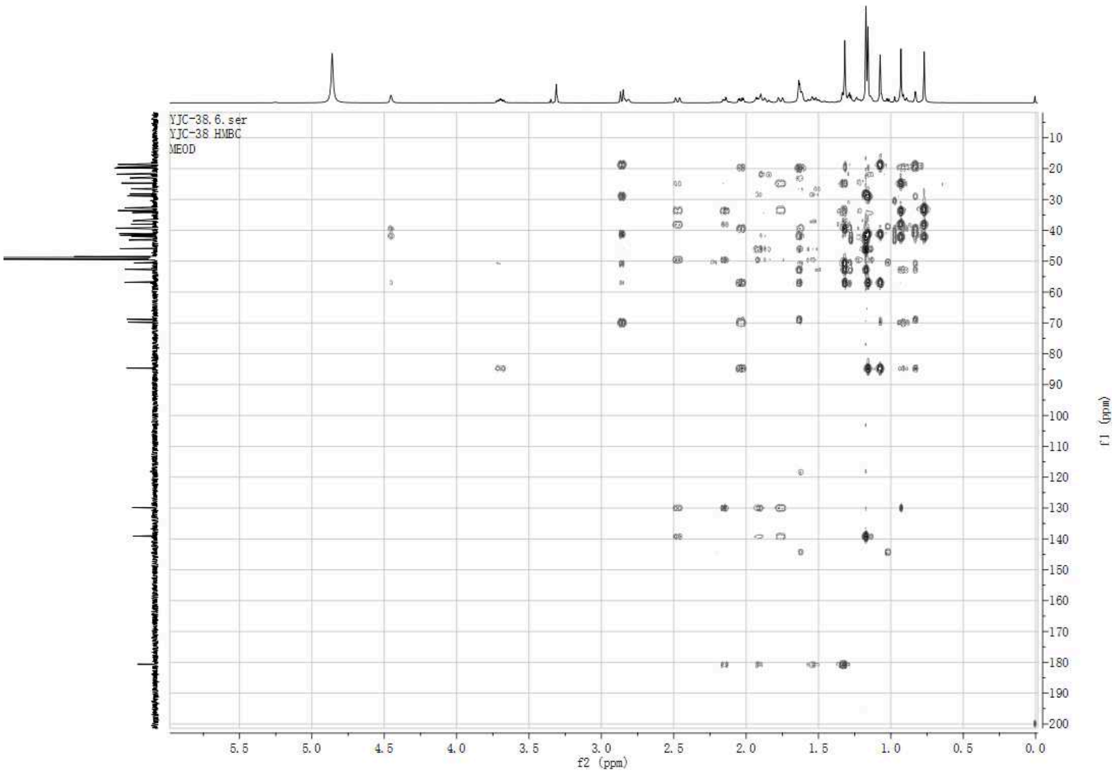

Figure S6. HMBC spectrum of **1**

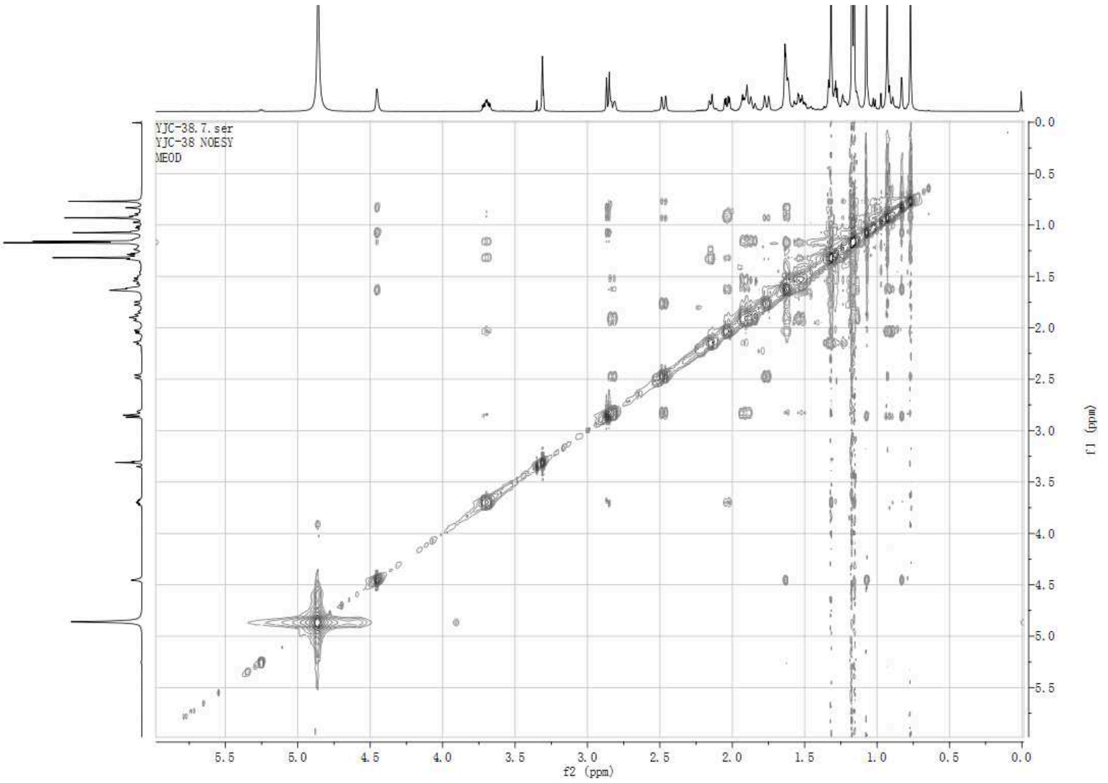

Figure S7. NOESY spectrum of **1**

Display Report

Analysis Info

Analysis Name	\\ESI-PC\data-D\Data\GJH\LJJ-YJC\20210506\YJC-38.d	Acquisition Date	2021/5/6 17:05:28
Method	tune_pos_standard_20141031.m	Operator	Demo User
Sample Name	YJC-38	Instrument	maXis HD 1820881.21303
Comment			

Acquisition Parameter

Source Type	ESI	Ion Polarity	Positive	Set Nebulizer	0.3 Bar
Focus	Active	Set Capillary	3500 V	Set Dry Heater	200 ¡ãC
Scan Begin	50 m/z	Set End Plate Offset	-500 V	Set Dry Gas	4.0 l/min
Scan End	3000 m/z	Set Charging Voltage	2000 V	Set Divert Valve	Waste
		Set Corona	0 nA	Set APCI Heater	0 ¡ãC

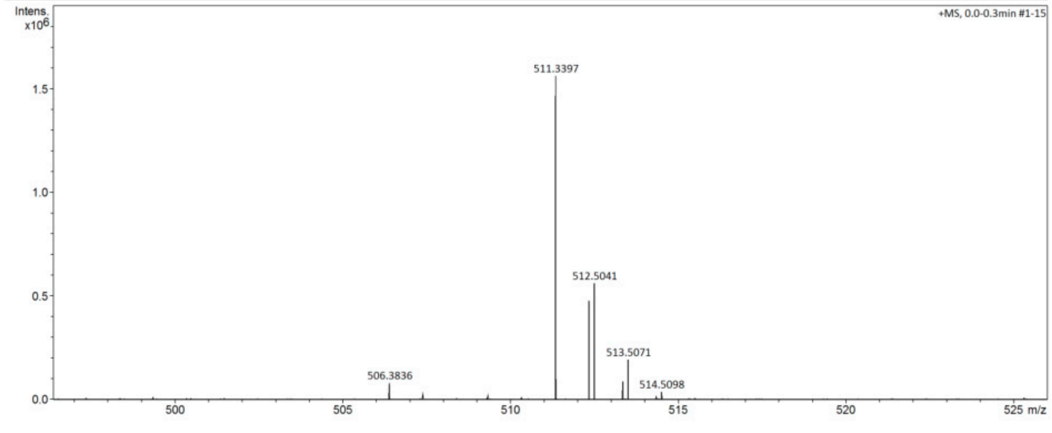

YJC-38.d

Bruker Compass DataAnalysis 4.2 printed: 2021/5/8 10:32:58 by: ZMH Page 1 of 1

Figure S8. HR-ESI-MS Spectrum of **1**

Thermo Scientific ~ VISIONpro SOFTWARE V4.41

Operator Name	(None Entered)	Date of Report	2021/10/9
Department	(None Entered)	Time of Report	22:07:01下午
Organization	(None Entered)		
Information	(None Entered)		

Scan Graph

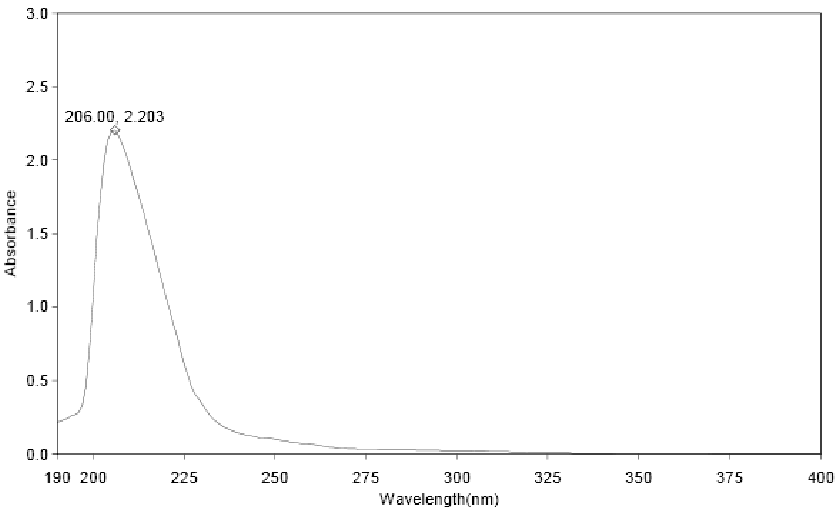

Results Table - scan021,YJC-38,Cycle01

nm	A	**Peak Pick Method**
206.00	2.203	Find 8 Peaks Above -3.0000 A
		Start Wavelength190.00 nm
		Stop Wavelength400.00 nm
		Sort By Wavelength

Sensitivity Auto

Figure S9. UV spectrum of **1**

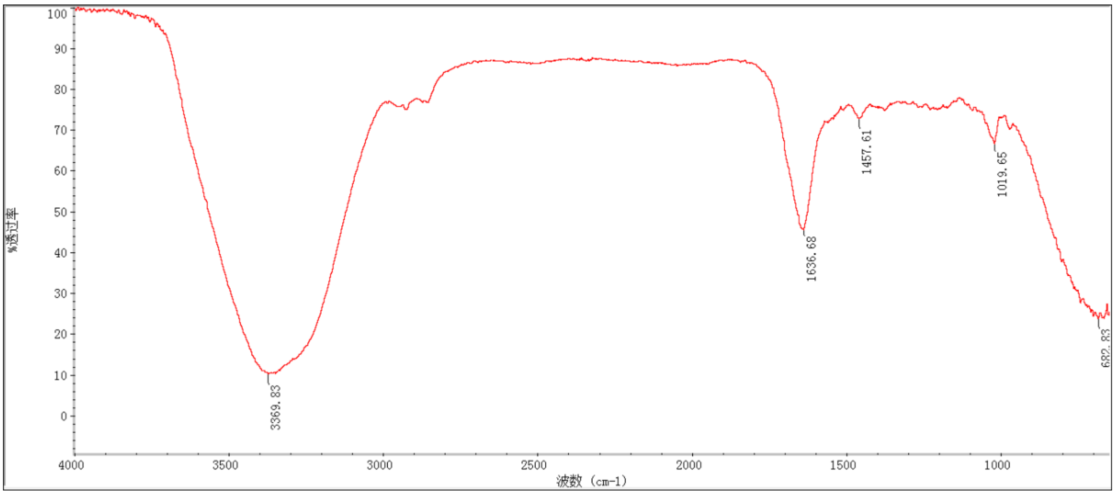

Figure S10. IR spectrum of **1**

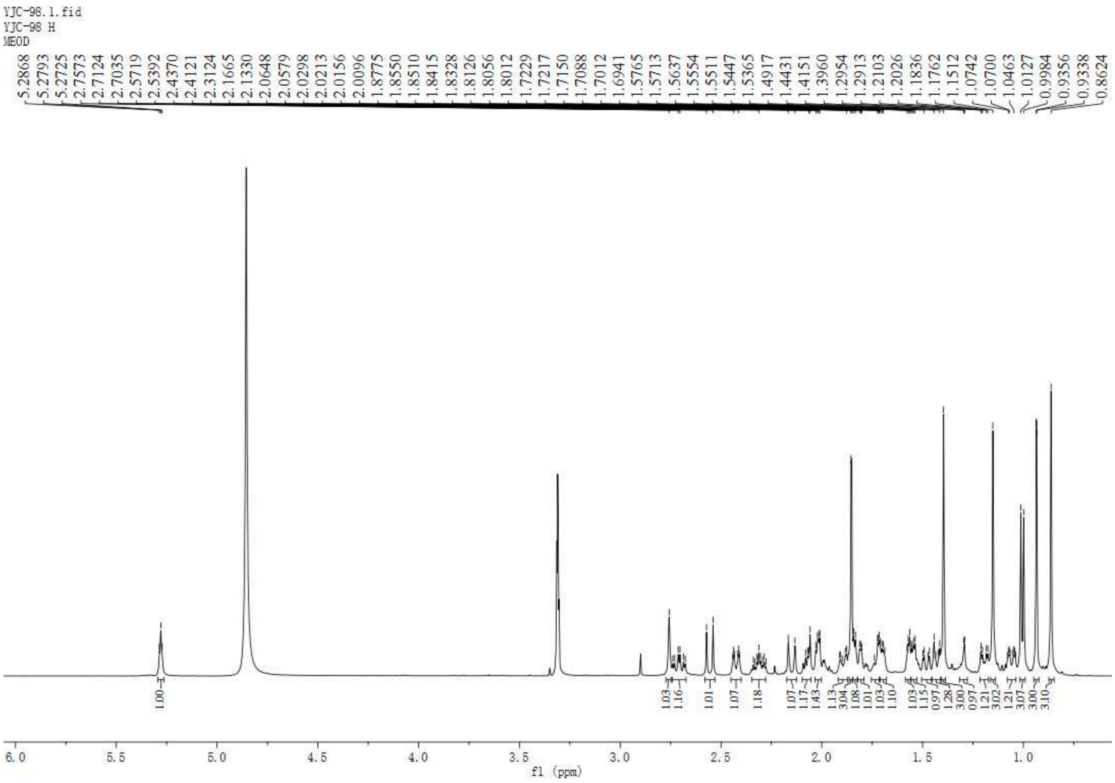

Figure S11. ^{1}H NMR spectrum (500 MHz, CD_3OD) of **2**

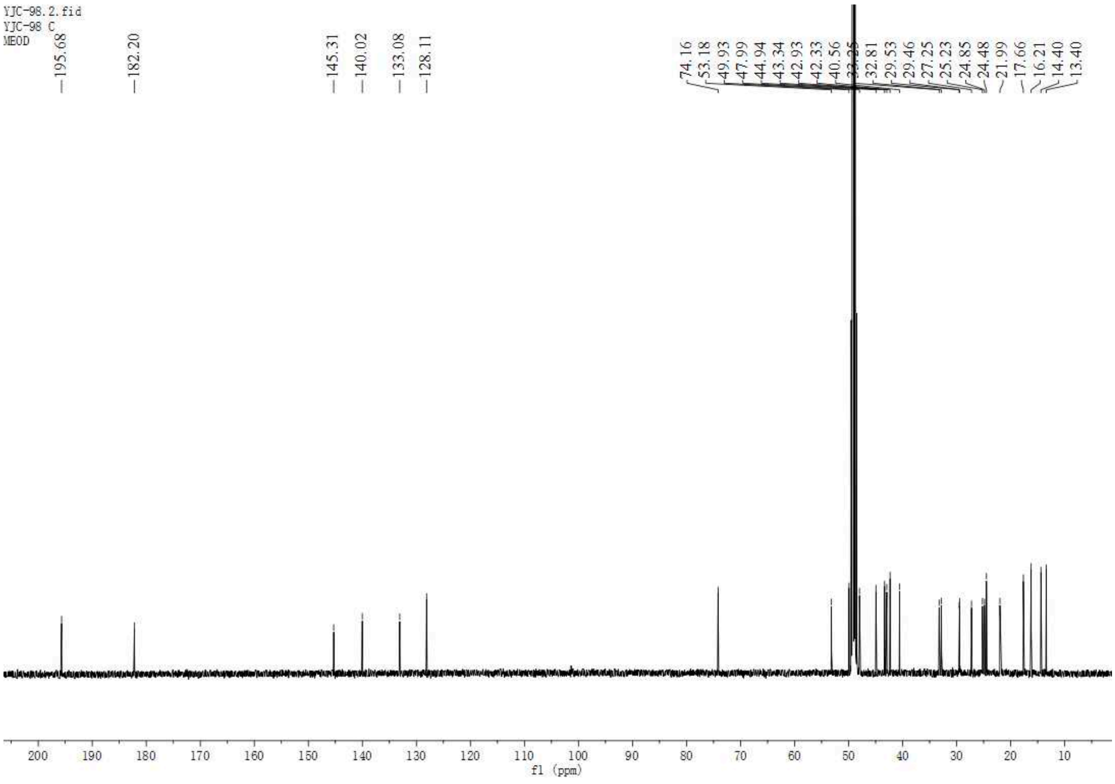

Figure S12. ^{13}C NMR spectrum (125 MHz, CD_3OD) of **2**

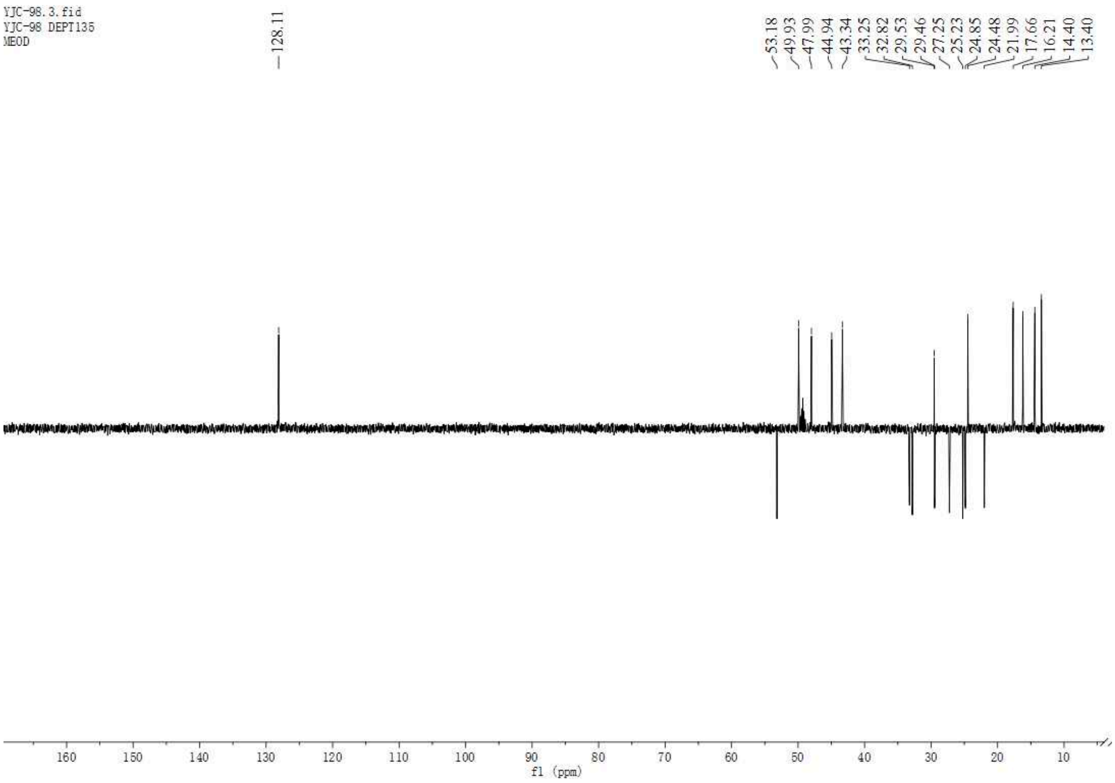

Figure S13. DEPT 135 spectrum (125 MHz, CD_3OD) of **2**

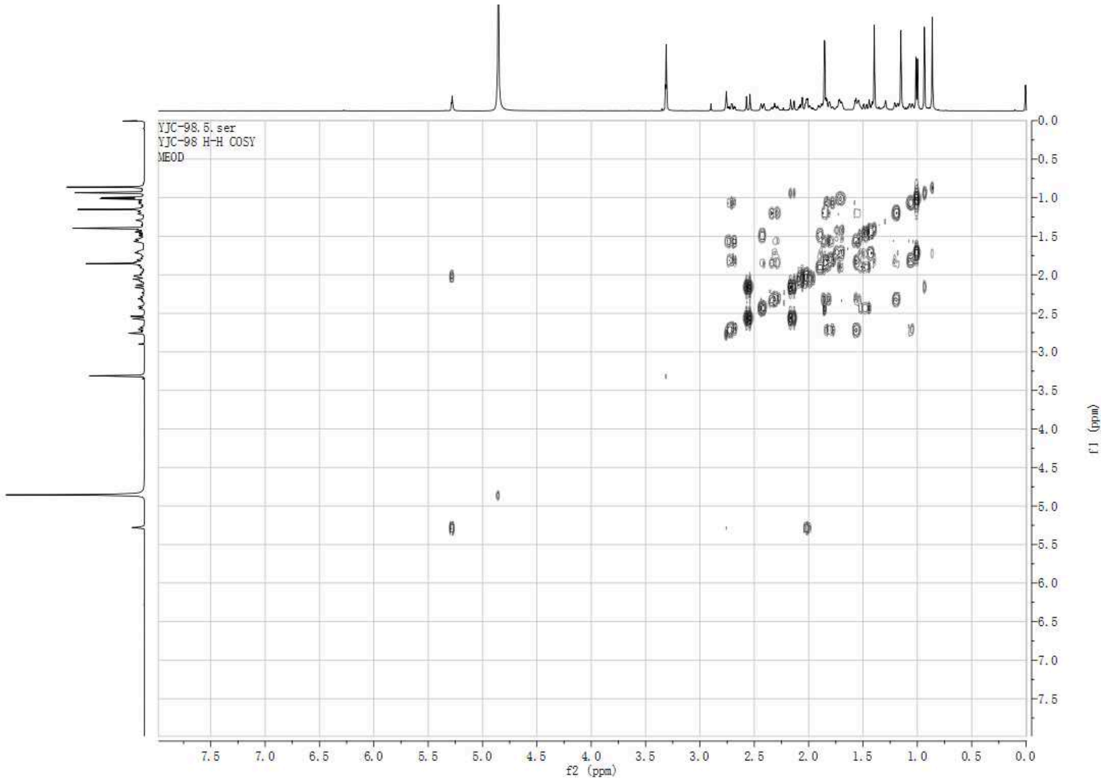

Figure S14. ^{1}H-^{1}H COSY spectrum of **2**

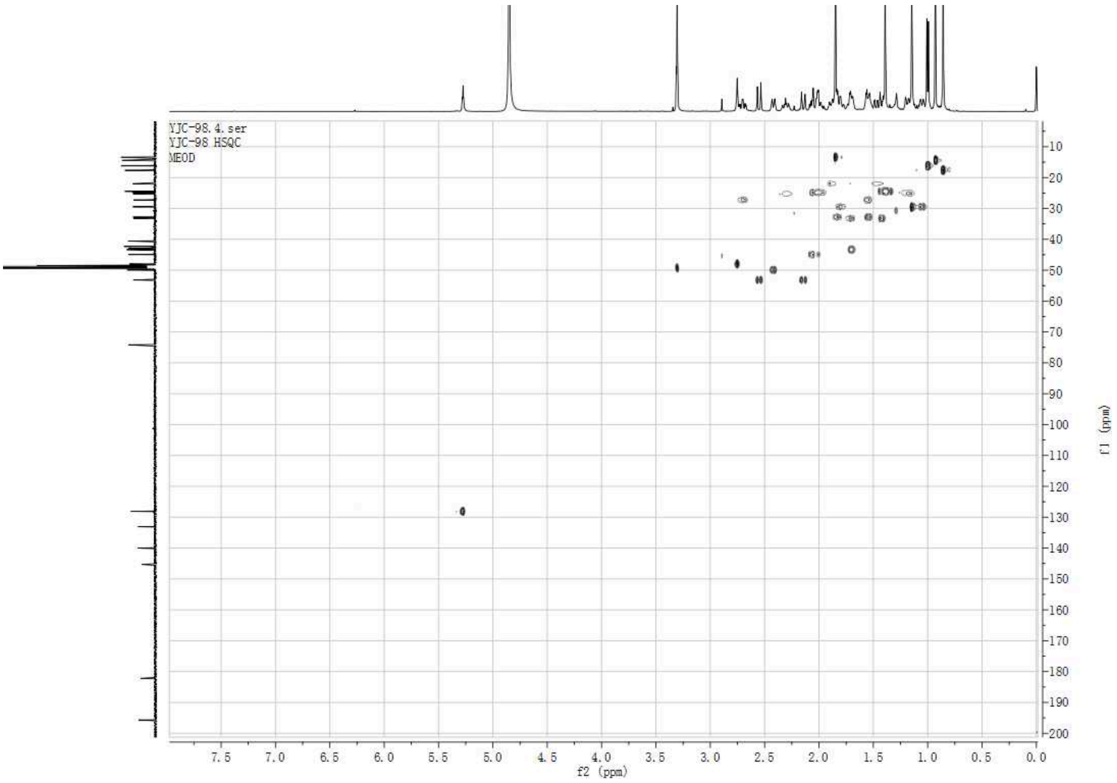

Figure S15. HSQC spectrum of **2**

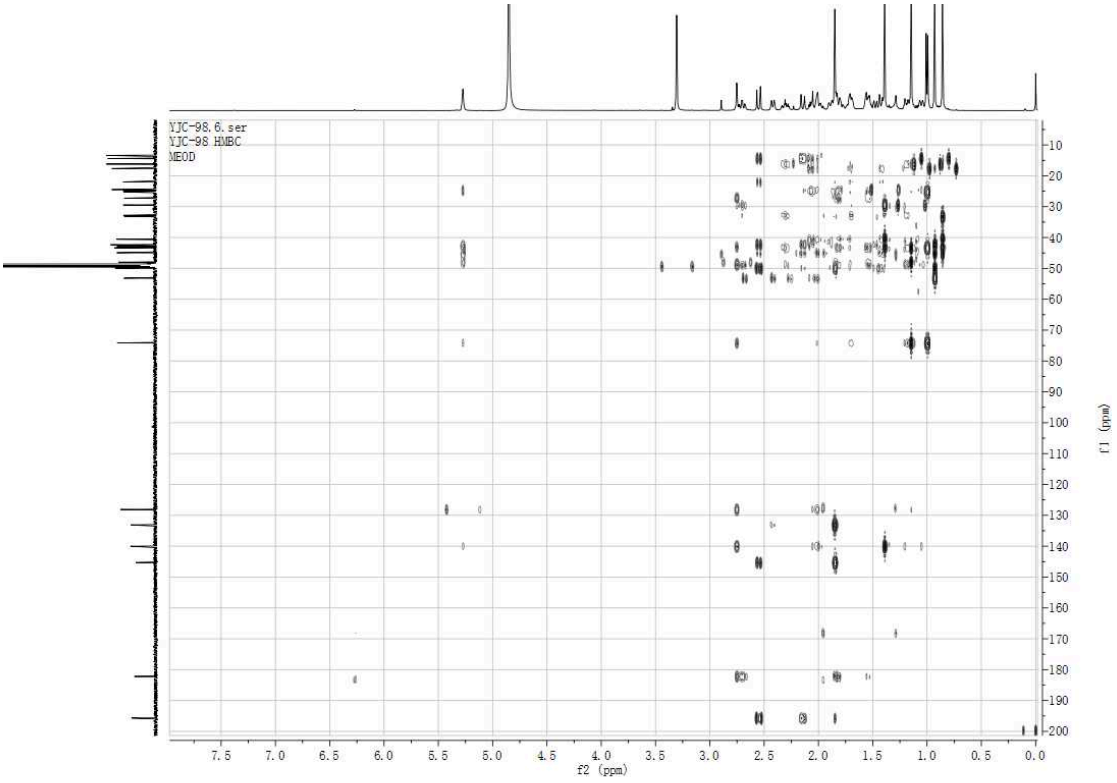

Figure S16. HMBC spectrum of **2**

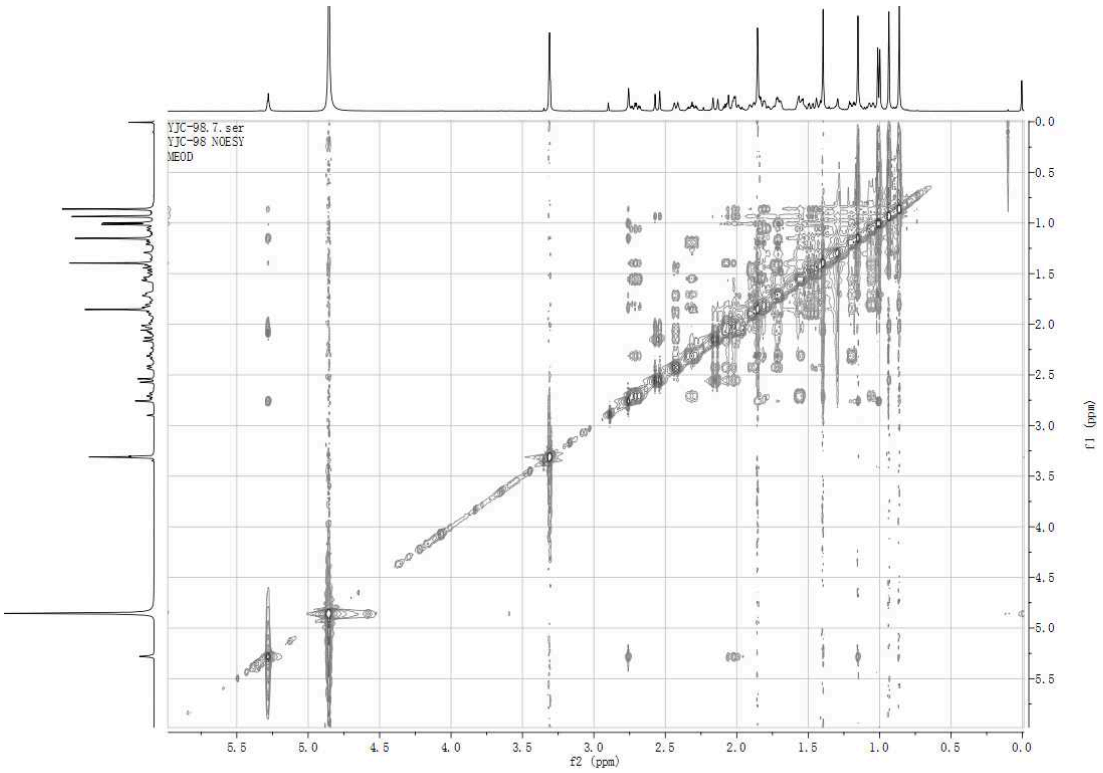

Figure S17. NOESY spectrum of **2**

Display Report

Analysis Info

Analysis Name	\\ESI-PC\data-D\Data\GJH\20211017-POS\YJC-98.d	Acquisition Date	2021/10/17 10:15:50
Method	tune_pos_standard_20141031.m	Operator	Demo User
Sample Name	YJC-98	Instrument	maXis HD 1820881.21303
Comment			

Acquisition Parameter

Source Type	ESI	Ion Polarity	Positive	Set Nebulizer	0.3 Bar
Focus	Active	Set Capillary	3500 V	Set Dry Heater	200 ¡ãC
Scan Begin	50 m/z	Set End Plate Offset	-500 V	Set Dry Gas	4.0 l/min
Scan End	3000 m/z	Set Charging Voltage	2000 V	Set Divert Valve	Waste
		Set Corona	0 nA	Set APCI Heater	0 ¡ãC

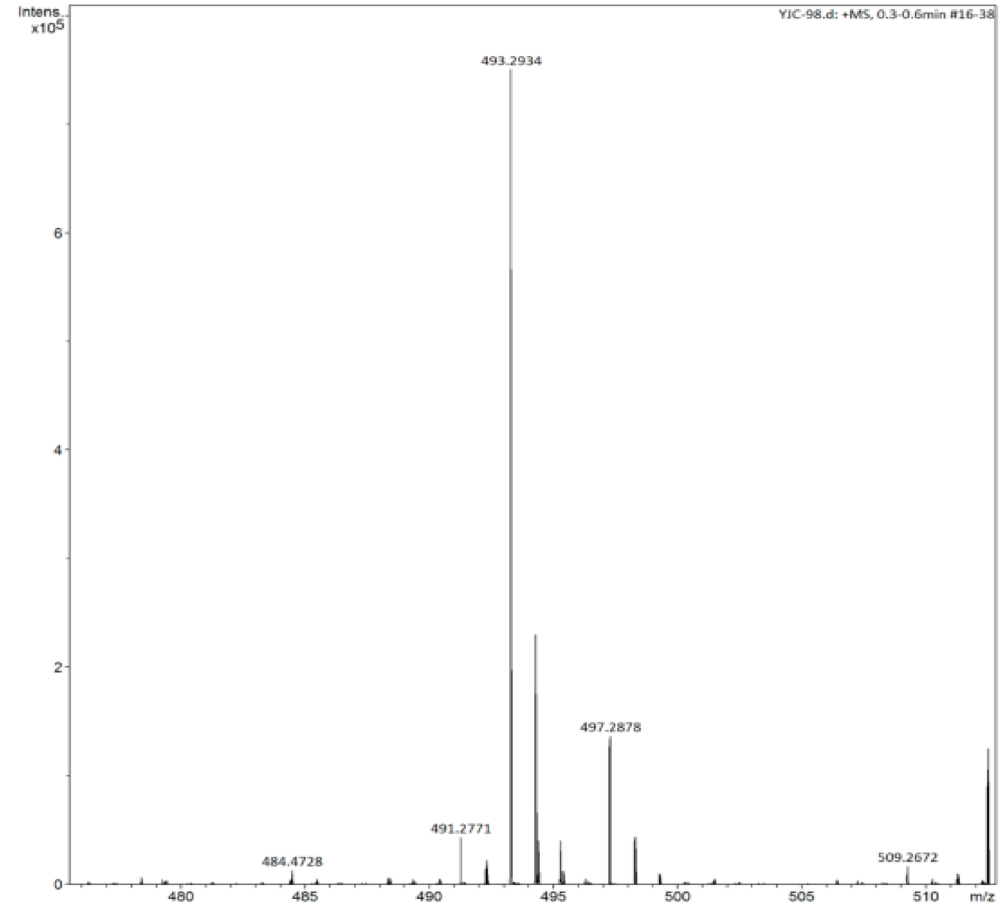

YJC-98.d

Bruker Compass DataAnalysis 4.2 printed: 2021/10/17 15:12:21 by: ZMH Page 1 of 1

Figure S18. HR-ESI-MS Spectrum of **2**

Thermo Scientific ~ VISIONpro SOFTWARE V4.41

Operator Name	(None Entered)	Date of Report	2021/10/9
Department	(None Entered)	Time of Report	22:11:18下午
Organization	(None Entered)		
Information	(None Entered)		

Scan Graph

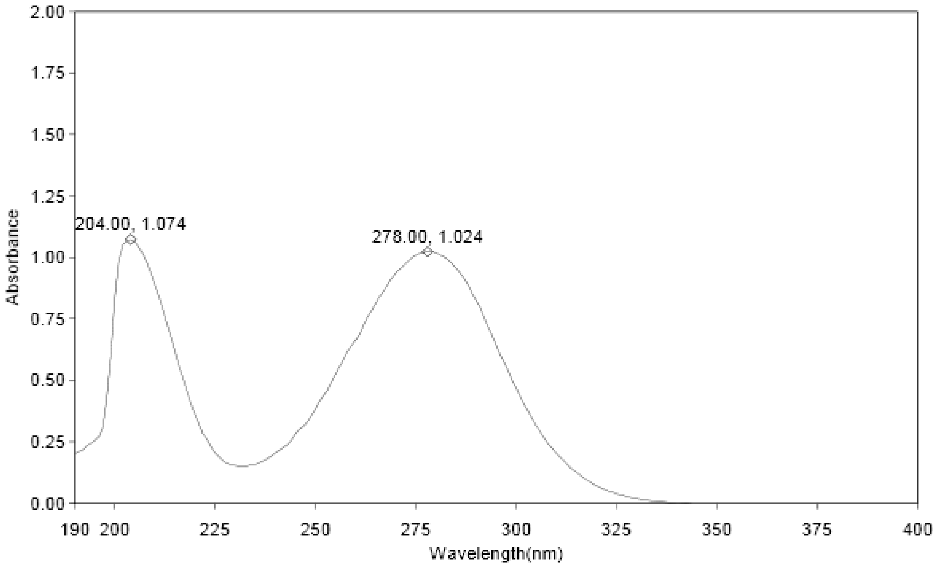

Results Table - scan022,YJC-98,Cycle01

nm	A	**Peak Pick Method**
204.00	1.074	Find 8 Peaks Above -3.0000 A
278.00	1.024	Start Wavelength190.00 nm
		Stop Wavelength400.00 nm
		Sort By Wavelength

Sensitivity Auto

Figure S19. UV spectrum of **2**

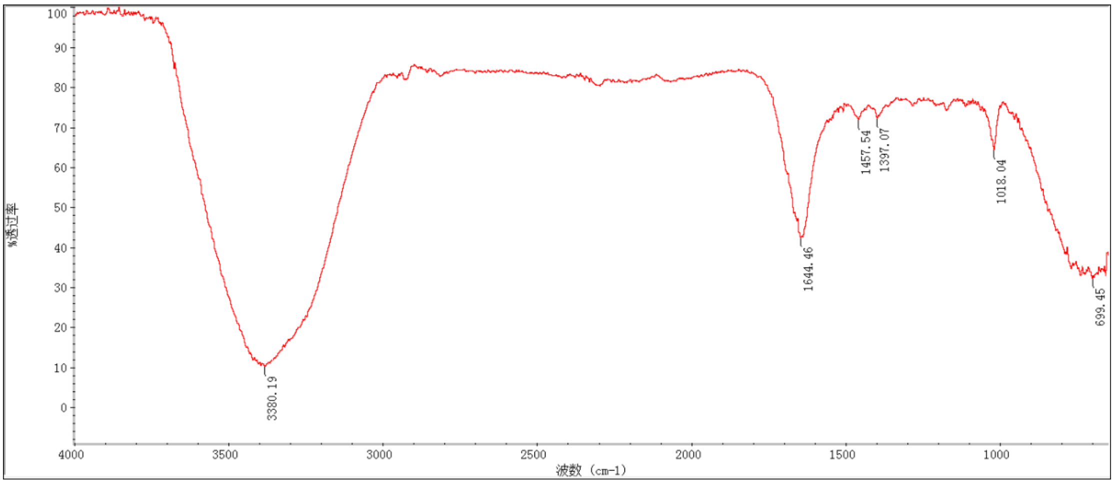

Figure S20. IR spectrum of **2**

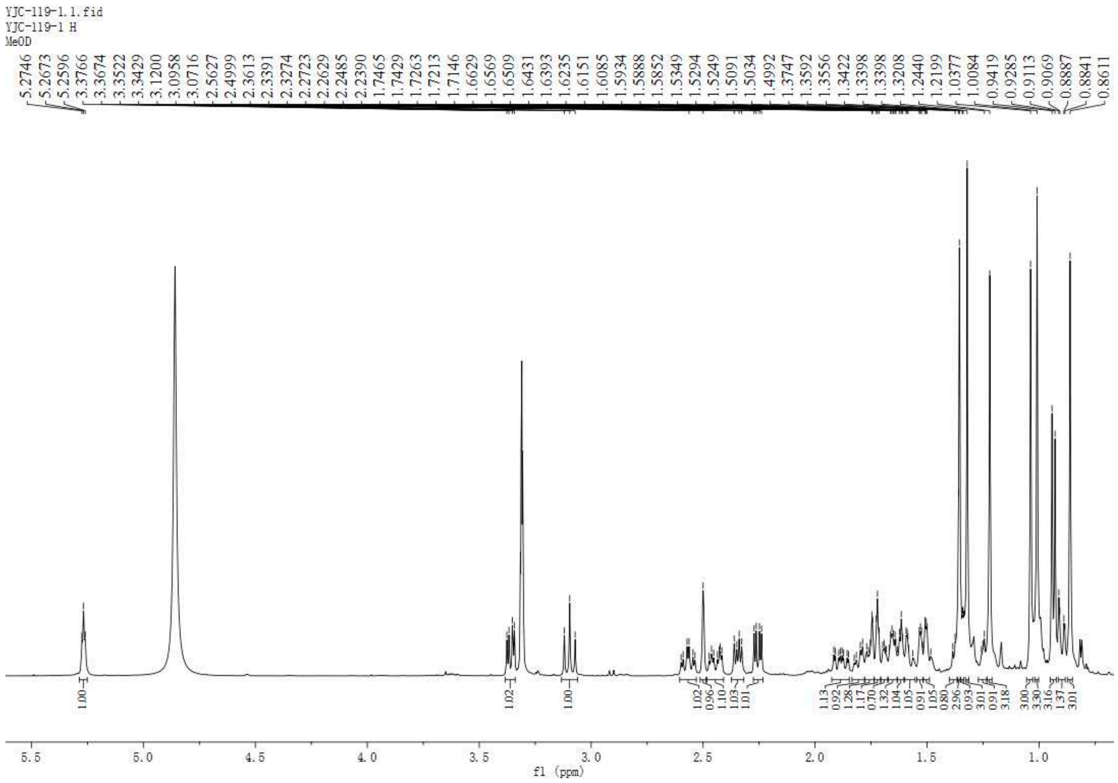

Figure S21. ^{1}H NMR spectrum (500 MHz, CD_3OD) of **3**

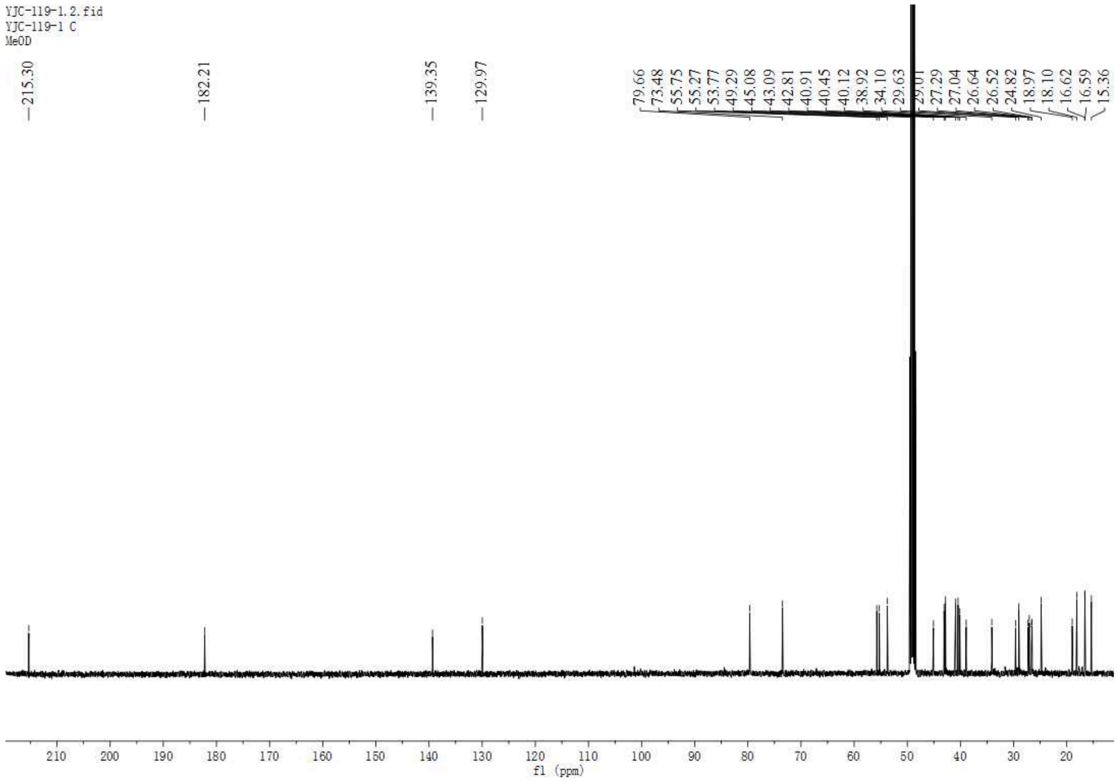

Figure S22. ^{13}C NMR spectrum (125 MHz, CD_3OD) of **3**

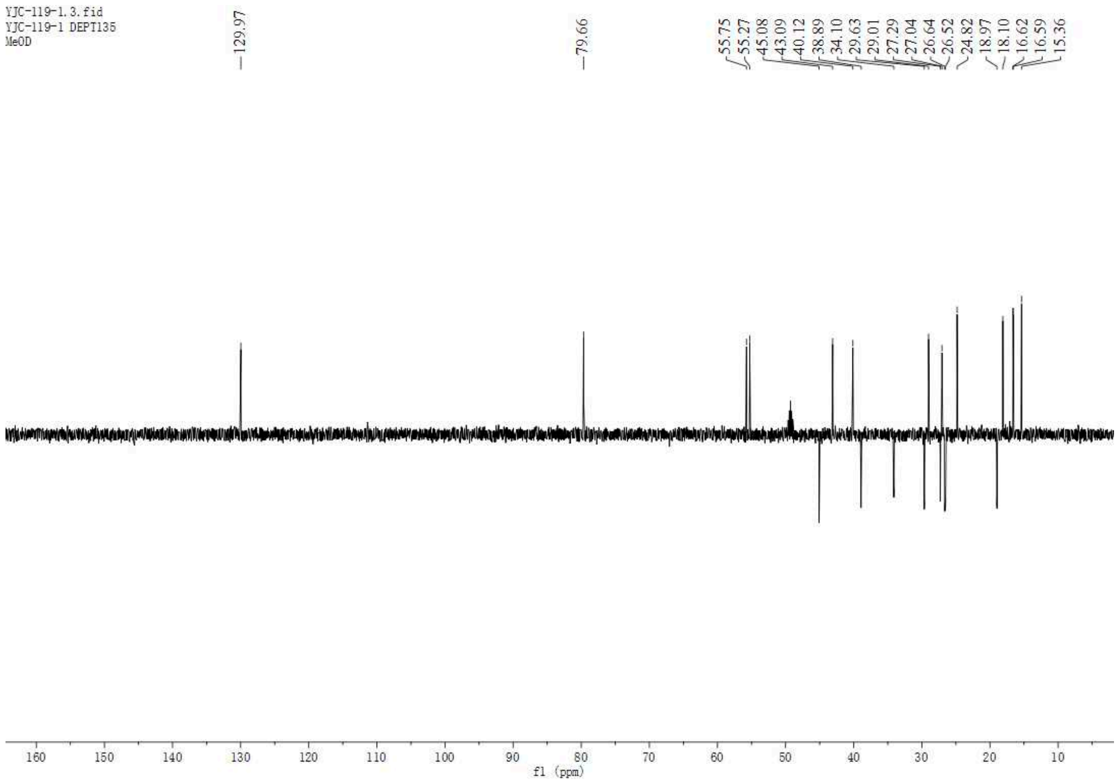

Figure S23. DEPT 135 spectrum (125 MHz, CD_3OD) of **3**

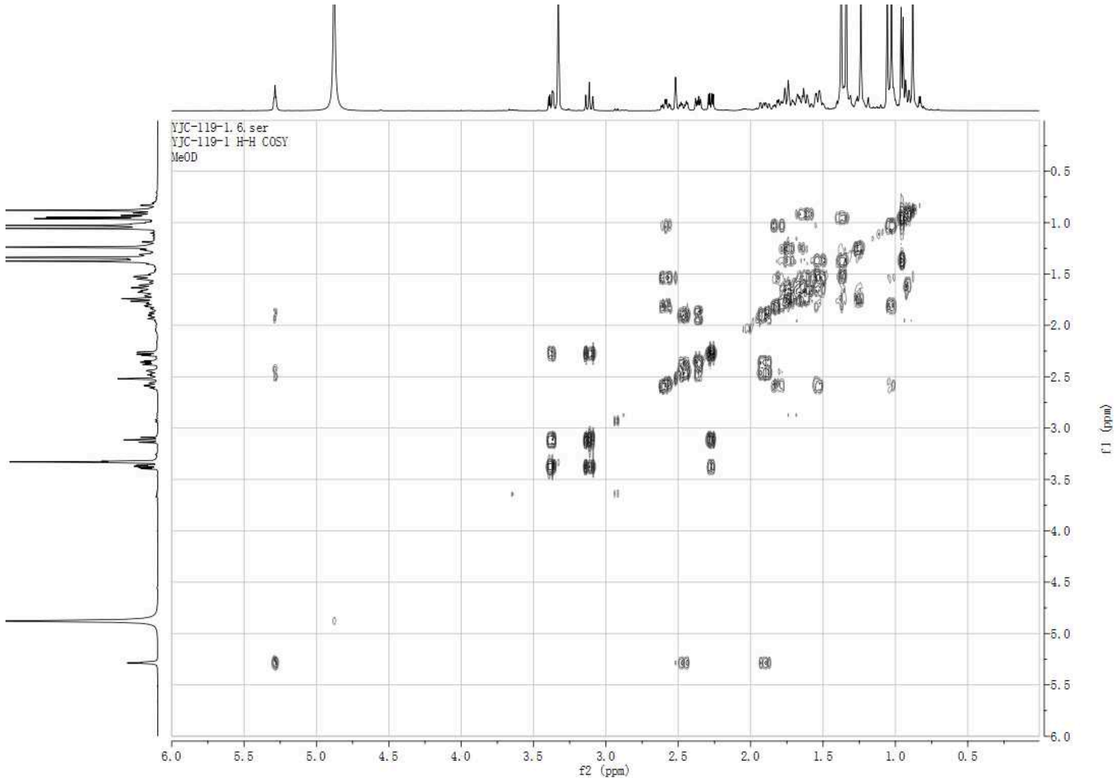

Figure S24. ^1H-^1H COSY spectrum of **3**

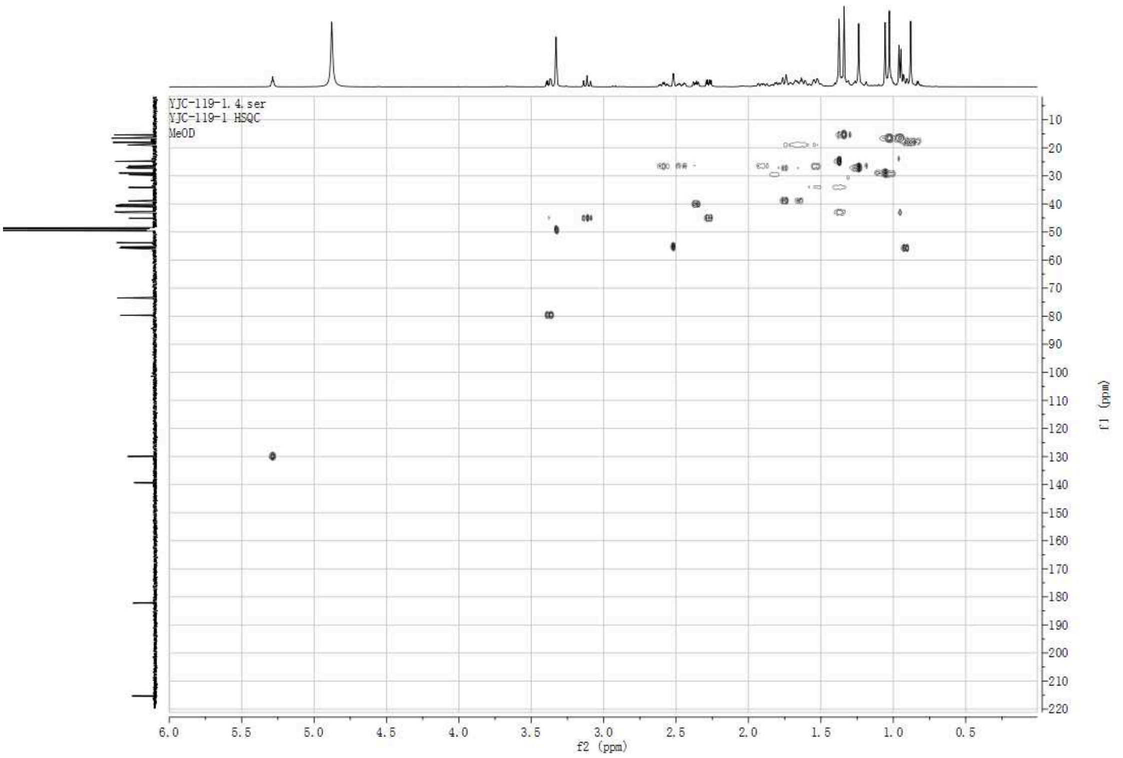

Figure S25. HSQC spectrum of **3**

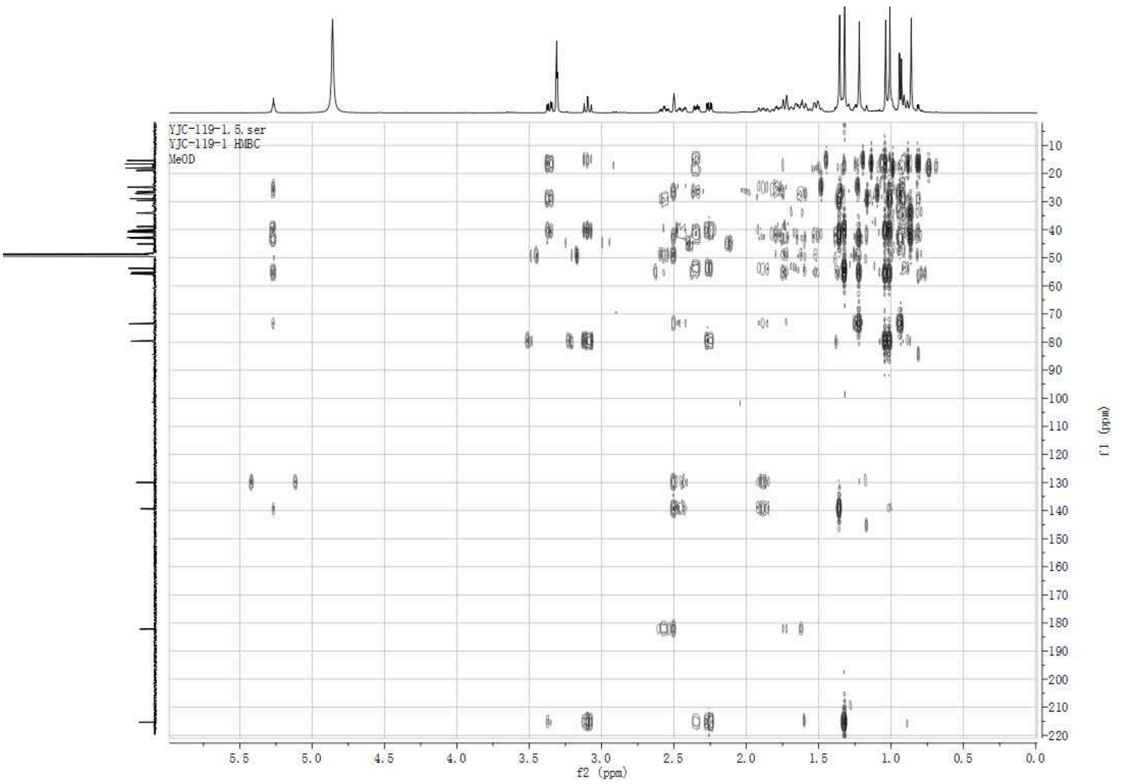

Figure S26. HMBC spectrum of **3**

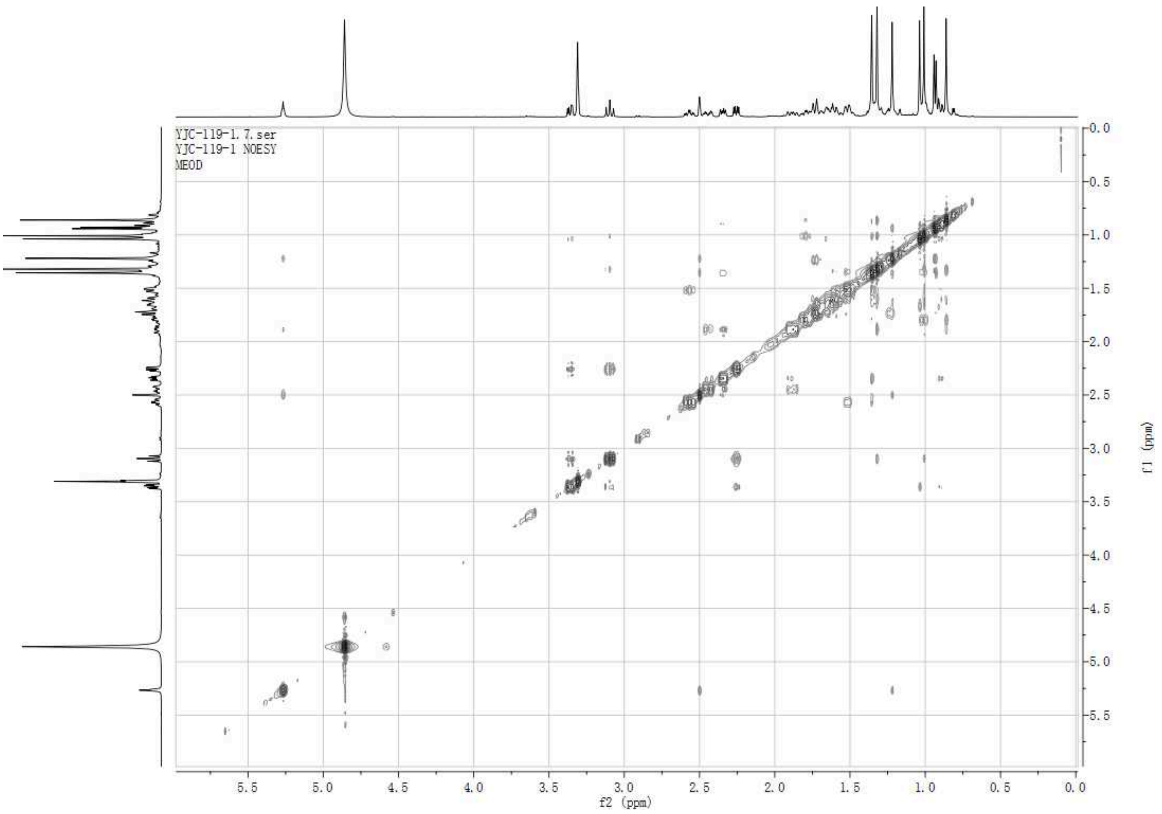

Figure S27. NOESY spectrum of **3**

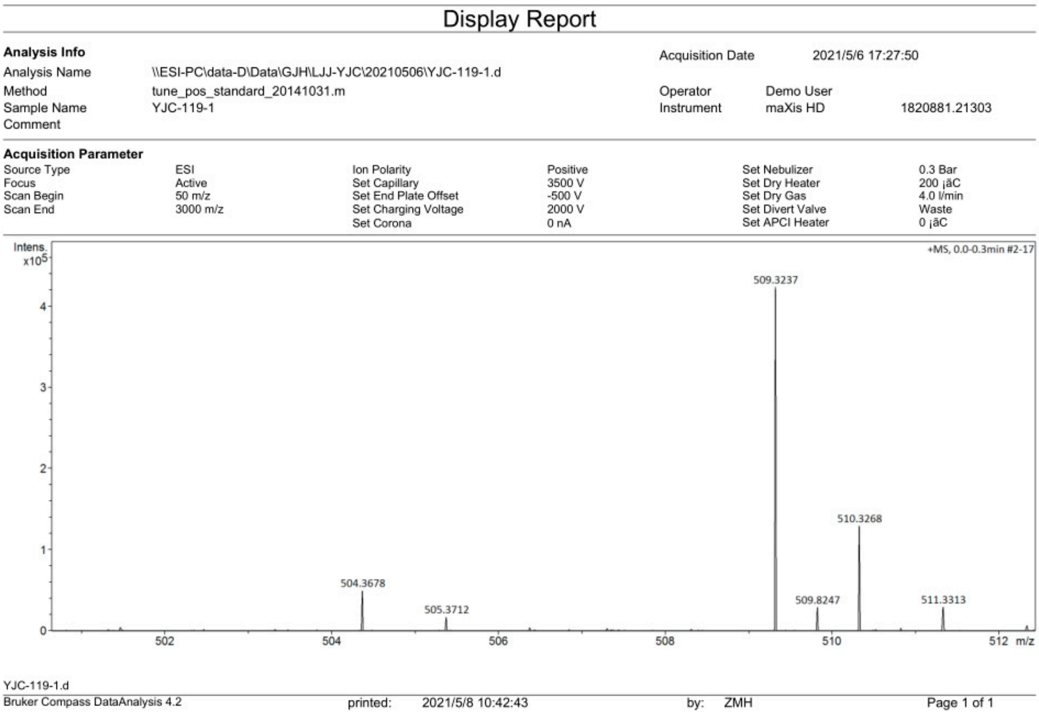

Figure S28. HR-ESI-MS Spectrum of **3**

Thermo Scientific ~ VISIONpro SOFTWARE V4.41

Operator Name	(None Entered)	Date of Report	2021/10/9
Department	(None Entered)	Time of Report	22:15:57下午
Organization	(None Entered)		
Information	(None Entered)		

Scan Graph

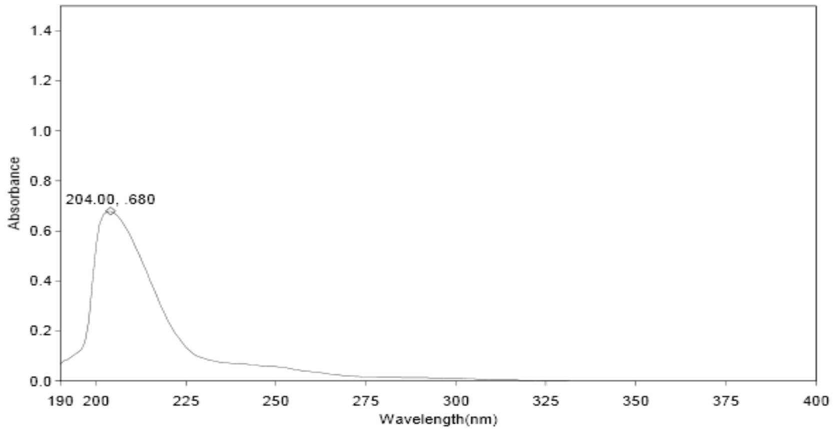

Results Table - scan023,YJC-119-1,Cycle01

nm	A	**Peak Pick Method**
204.00	.680	Find 8 Peaks Above -3.0000 A
		Start Wavelength190.00 nm
		Stop Wavelength400.00 nm
		Sort By Wavelength

Sensitivity Auto

Figure S29. UV spectrum of **3**

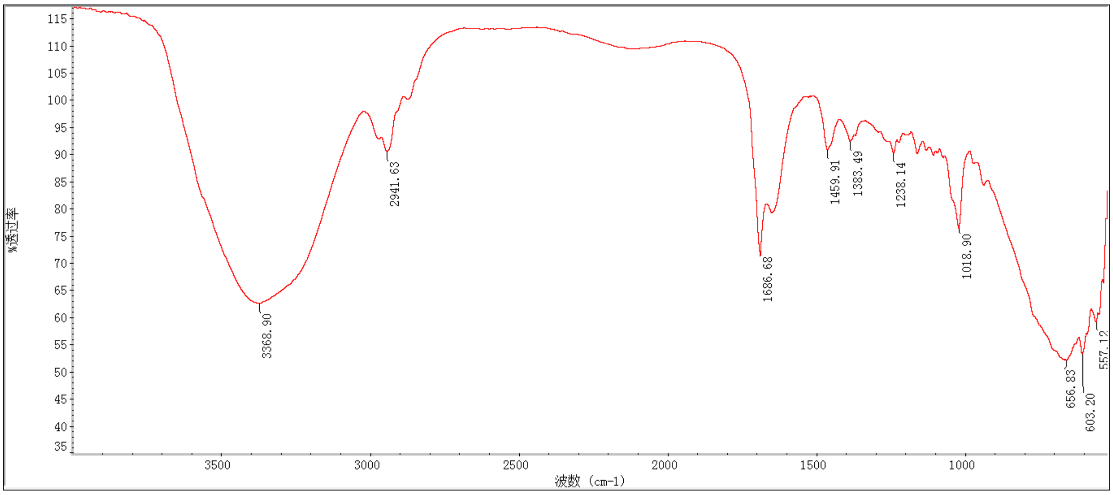

Figure S30. IR spectrum of **3**

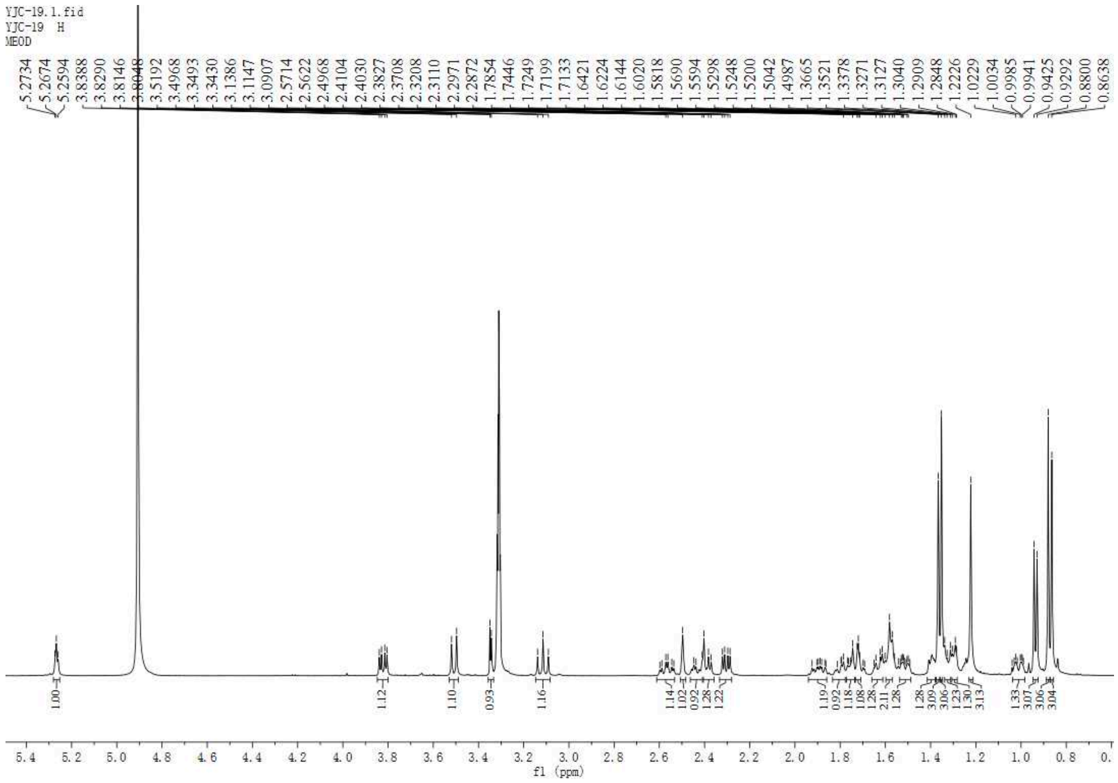

Figure S31. ^{1}H NMR spectrum (500 MHz, CD_3OD) of **4**

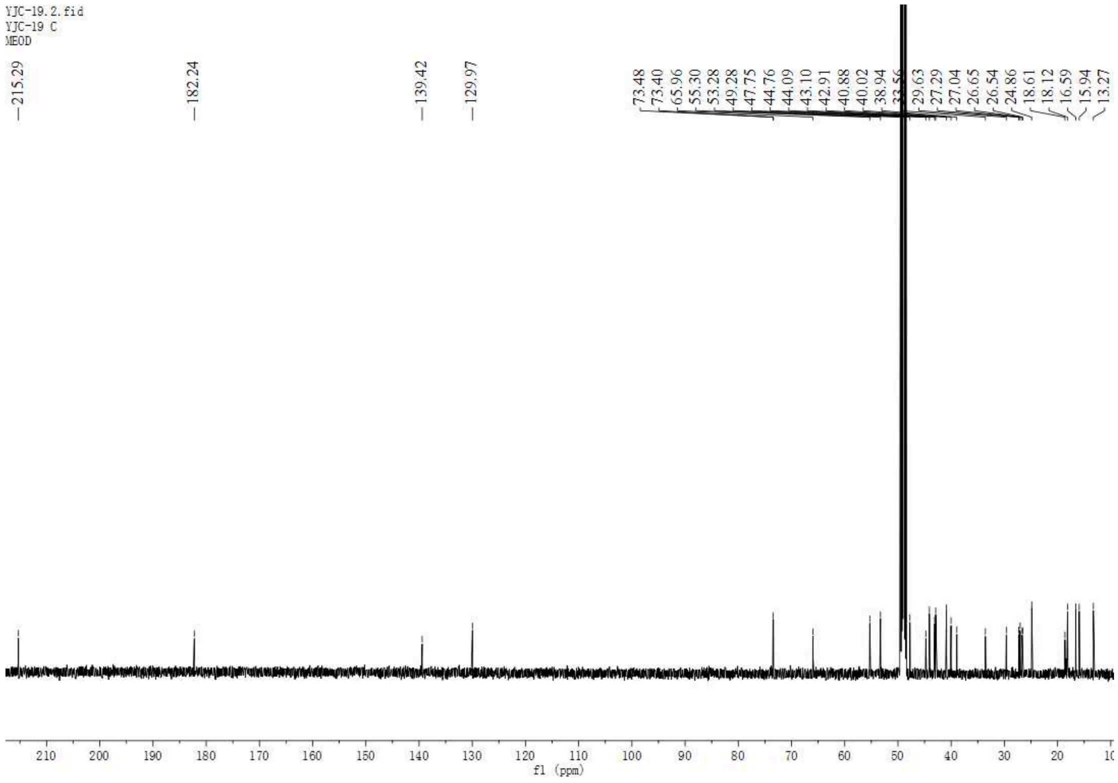

Figure S32. ^{13}C NMR spectrum (125 MHz, CD_3OD) of **4**

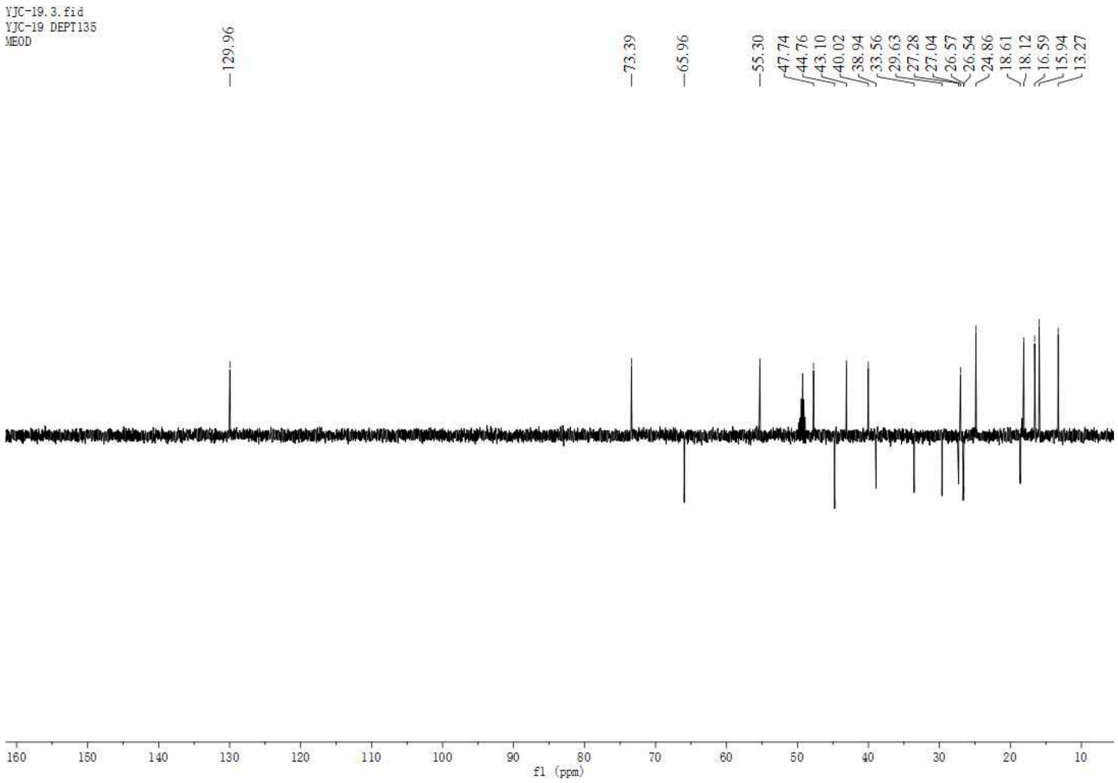

Figure S33. DEPT 135 spectrum (125 MHz, CD_3OD) of **4**

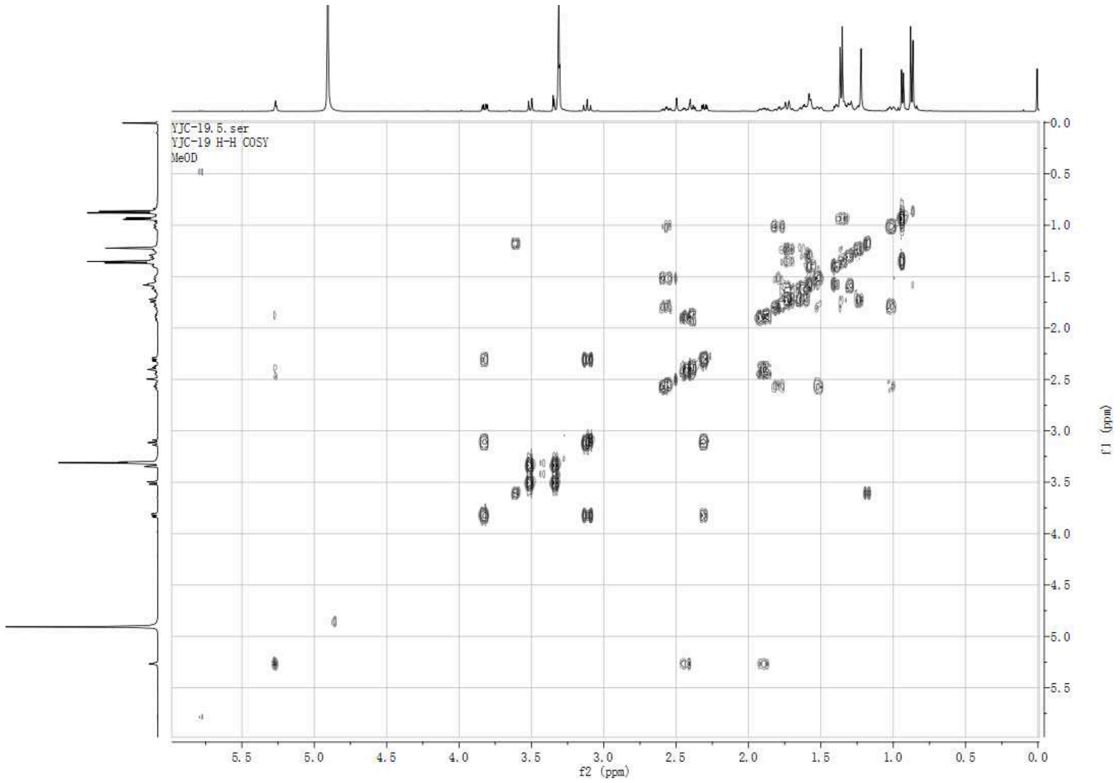

Figure S34. ^1H-^1H COSY spectrum of **4**

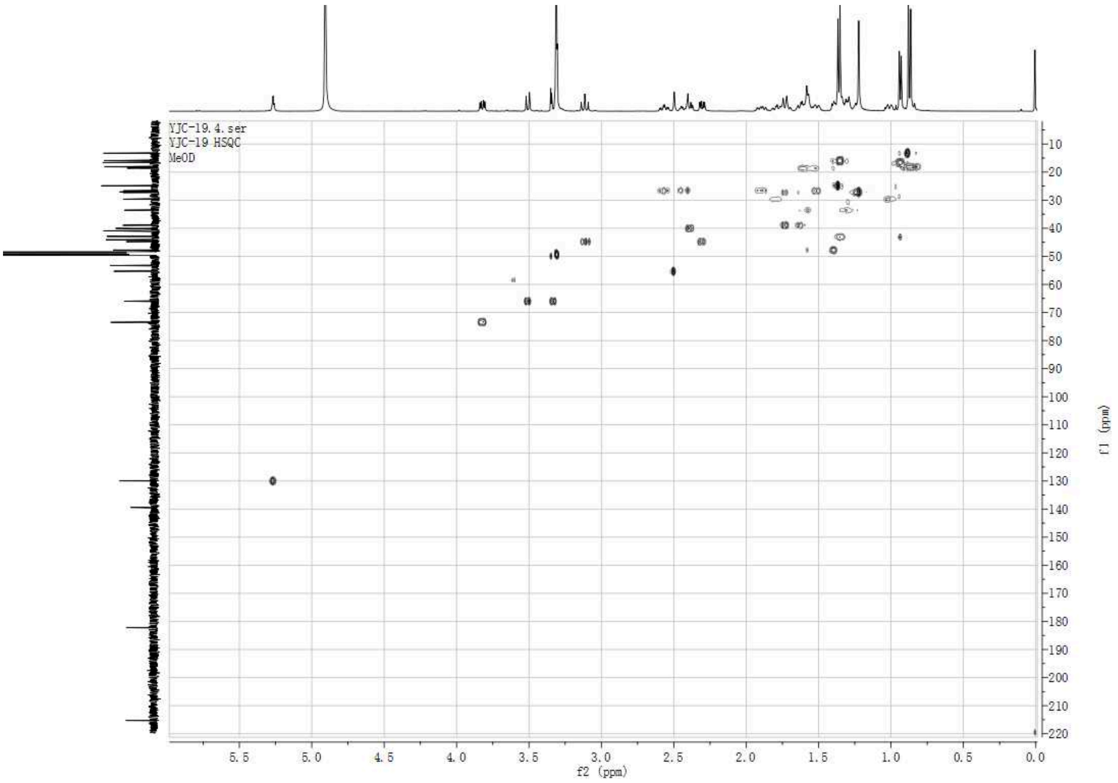

Figure S35. HSQC spectrum of **4**

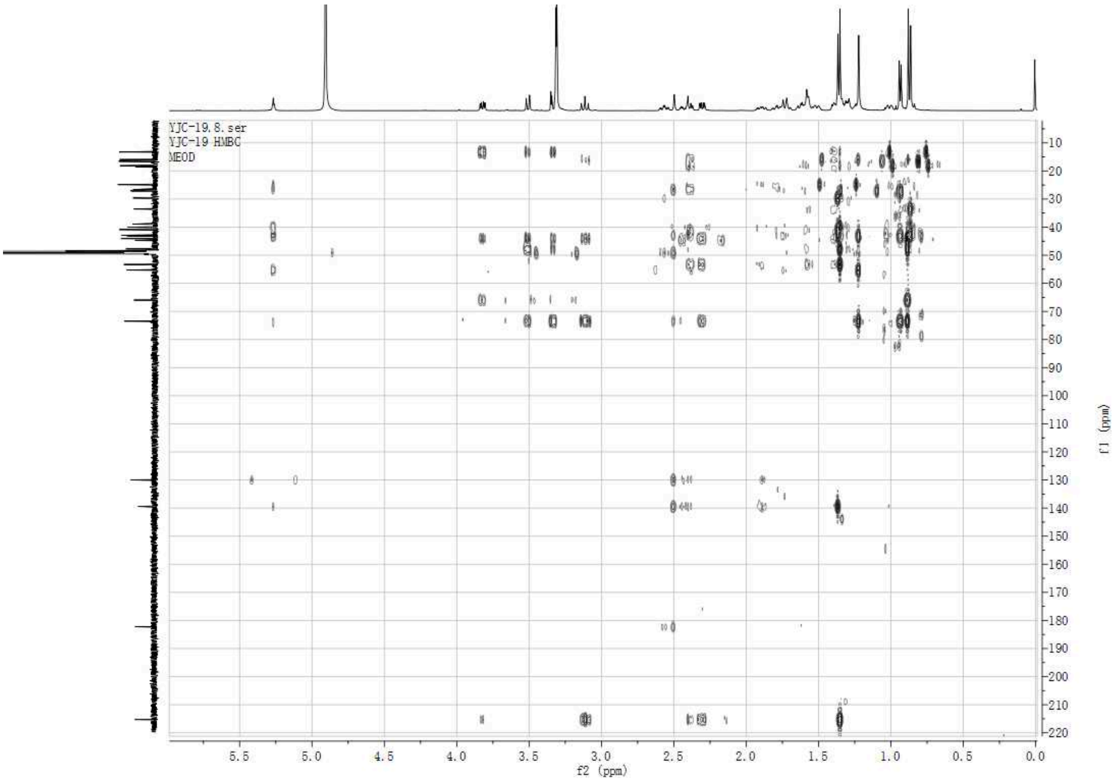

Figure S36. HMBC spectrum of **4**

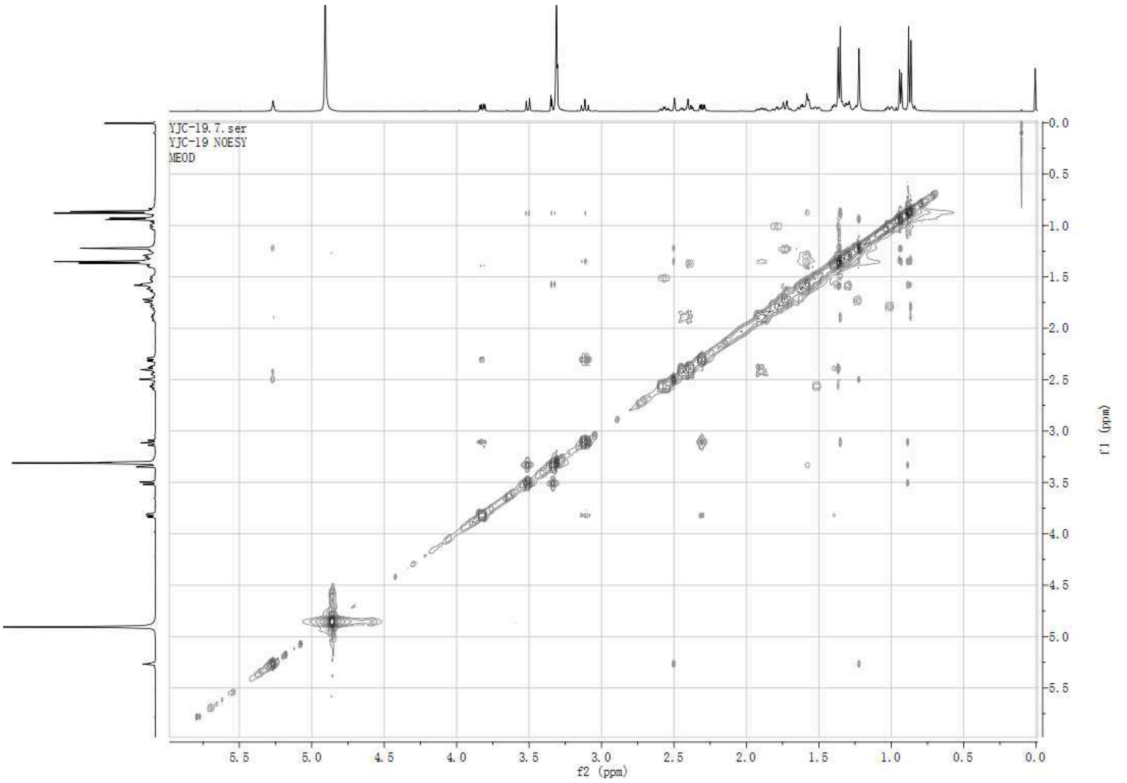

Figure S37. NOESY spectrum of **4**

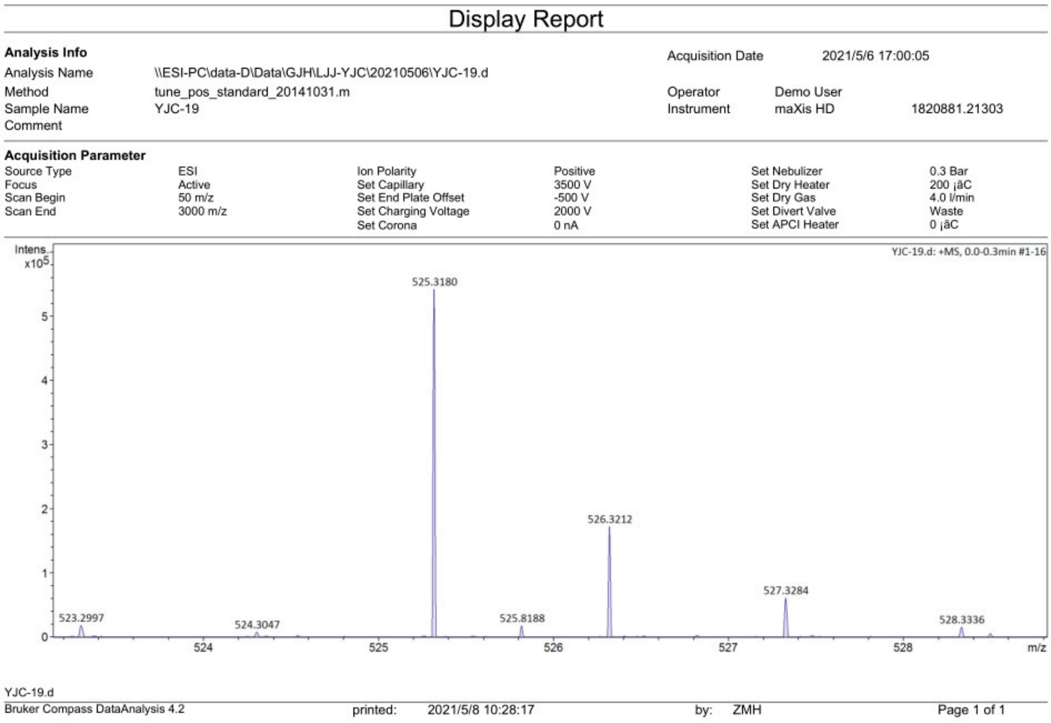

Figure S38. HR-ESI-MS Spectrum of **4**

Thermo Scientific ~ VISIONpro SOFTWARE V4.41

Operator Name	(None Entered)	Date of Report	2021/10/9
Department	(None Entered)	Time of Report	22:01:46下午
Organization	(None Entered)		
Information	(None Entered)		

Scan Graph

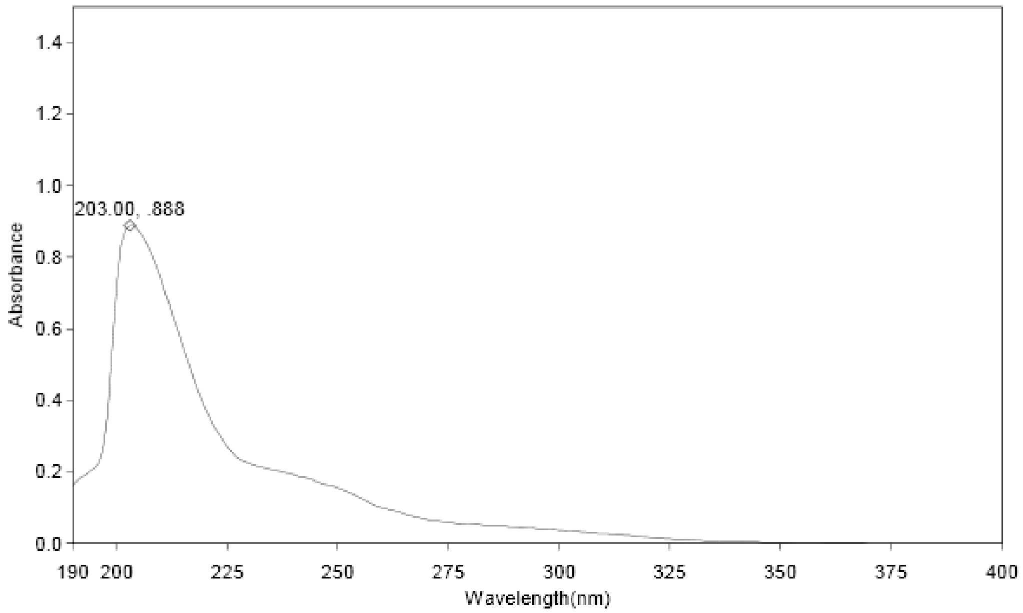

Results Table - scan020,YJC-19,Cycle01

nm	A	Peak Pick Method
203.00	.888	Find 8 Peaks Above -3.0000 A
		Start Wavelength190.00 nm
		Stop Wavelength400.00 nm
		Sort By Wavelength

Sensitivity Auto

Figure S39. UV spectrum of **4**

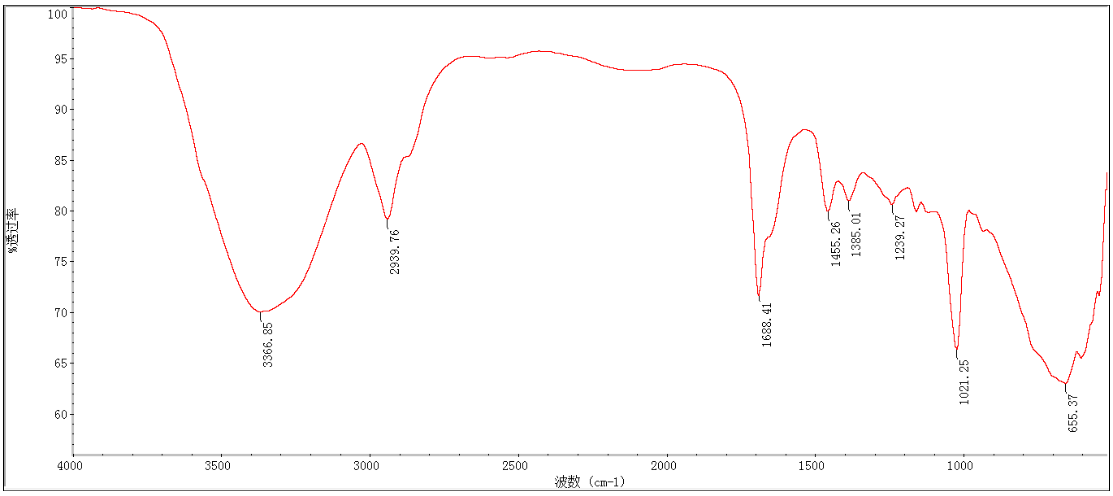

Figure S40. IR spectrum of **4**

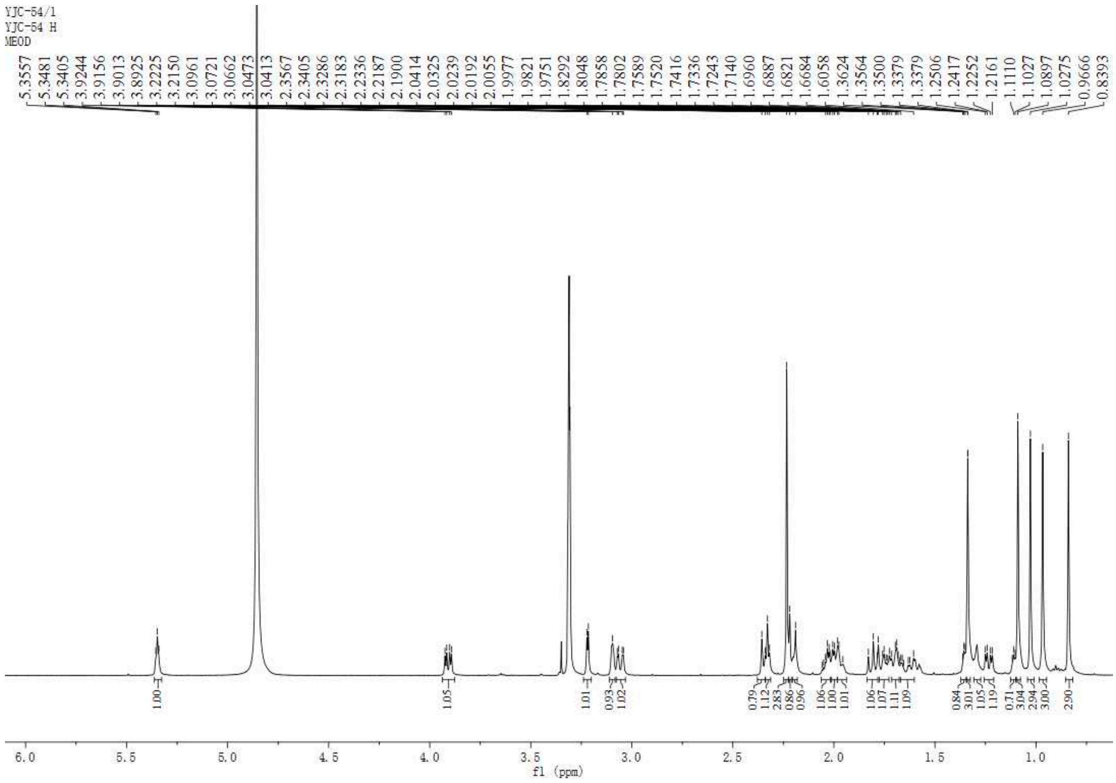

Figure S41. ^{1}H NMR spectrum (500 MHz, CD_3OD) of **5**

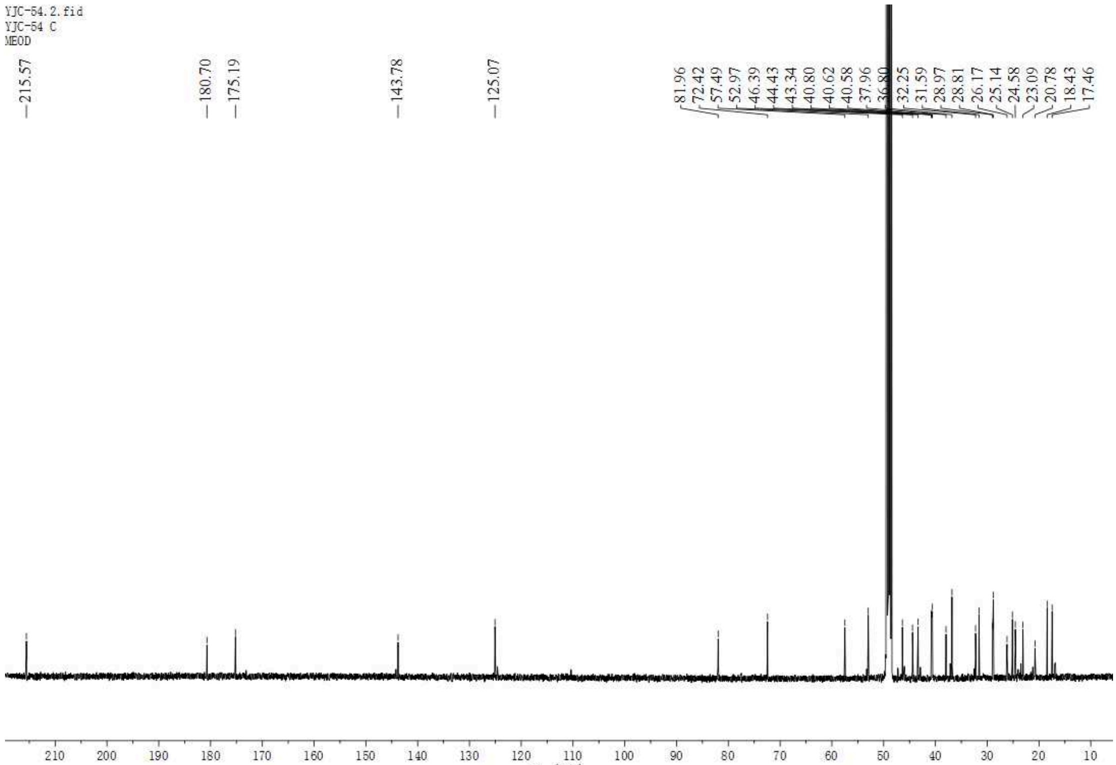

Figure S42. ^{13}C NMR spectrum (125 MHz, CD_3OD) of **5**

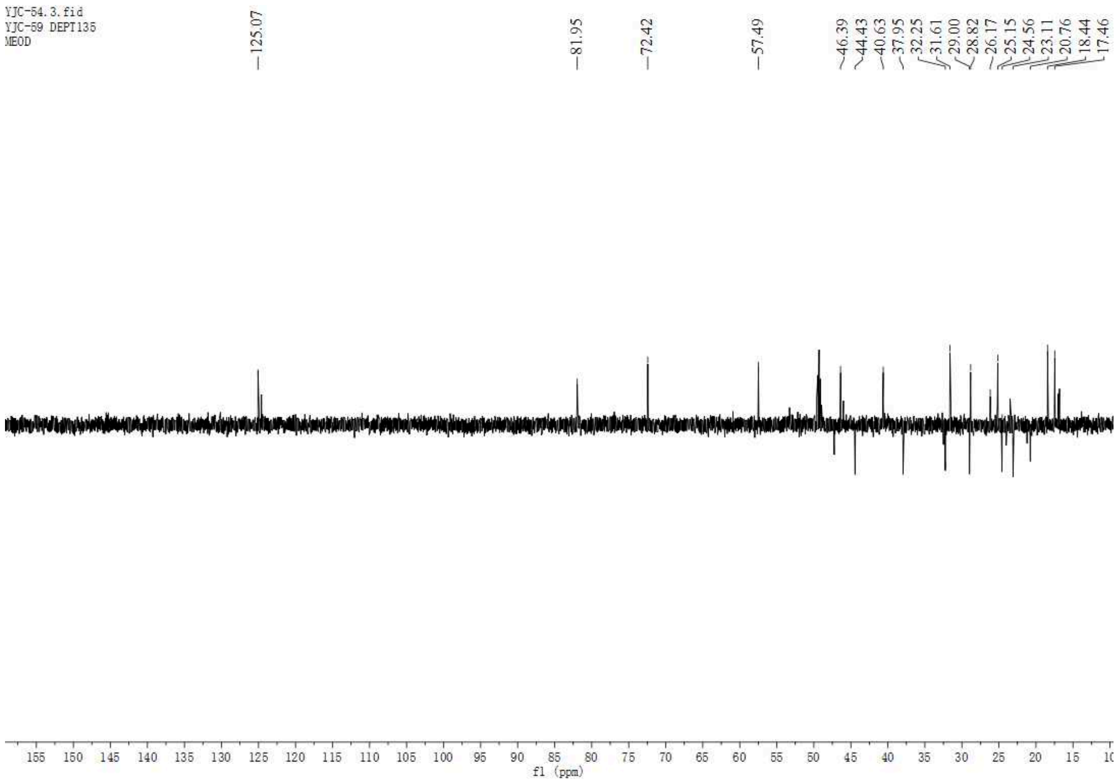

Figure S43. DEPT 135 spectrum (125 MHz, CD_3OD) of **5**

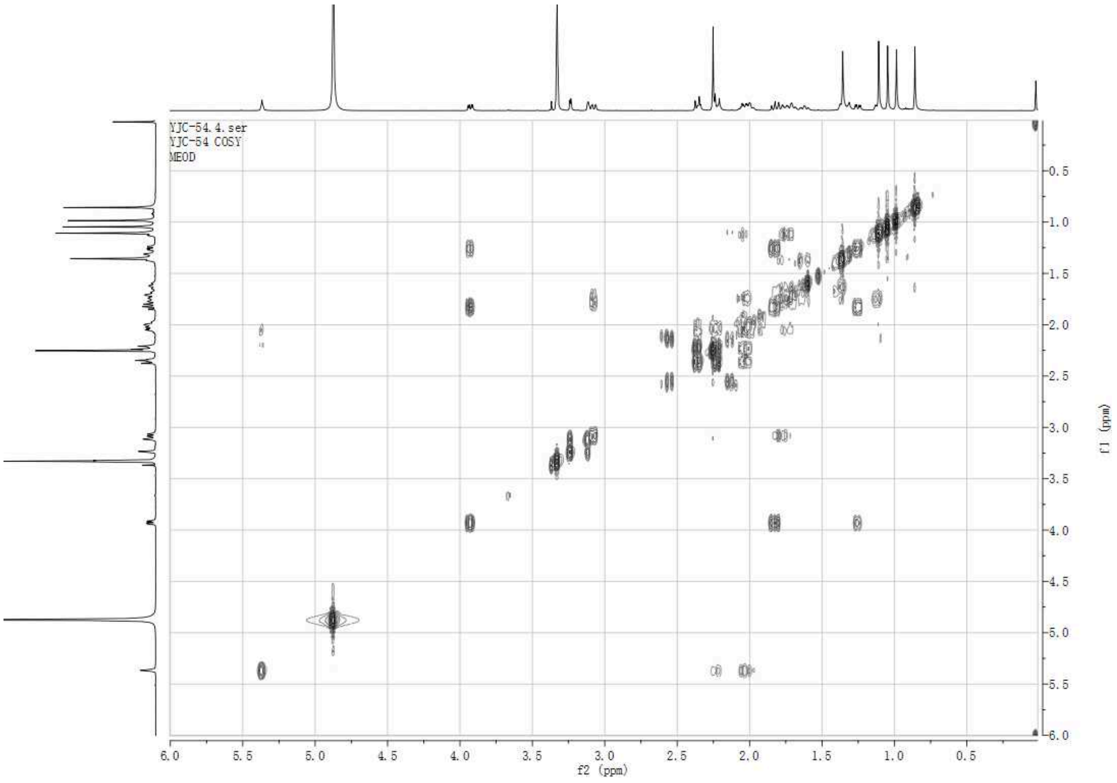

Figure S44. ^1H-^1H COSY spectrum of **5**

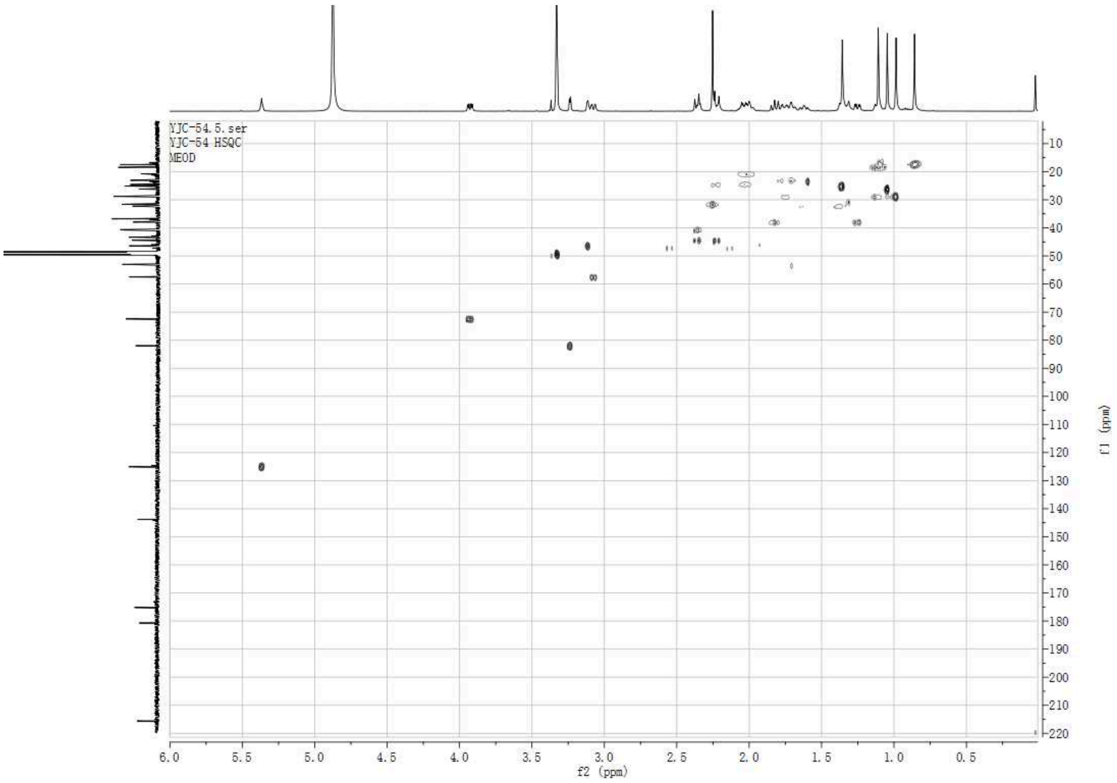

Figure S45. HSQC spectrum of **5**

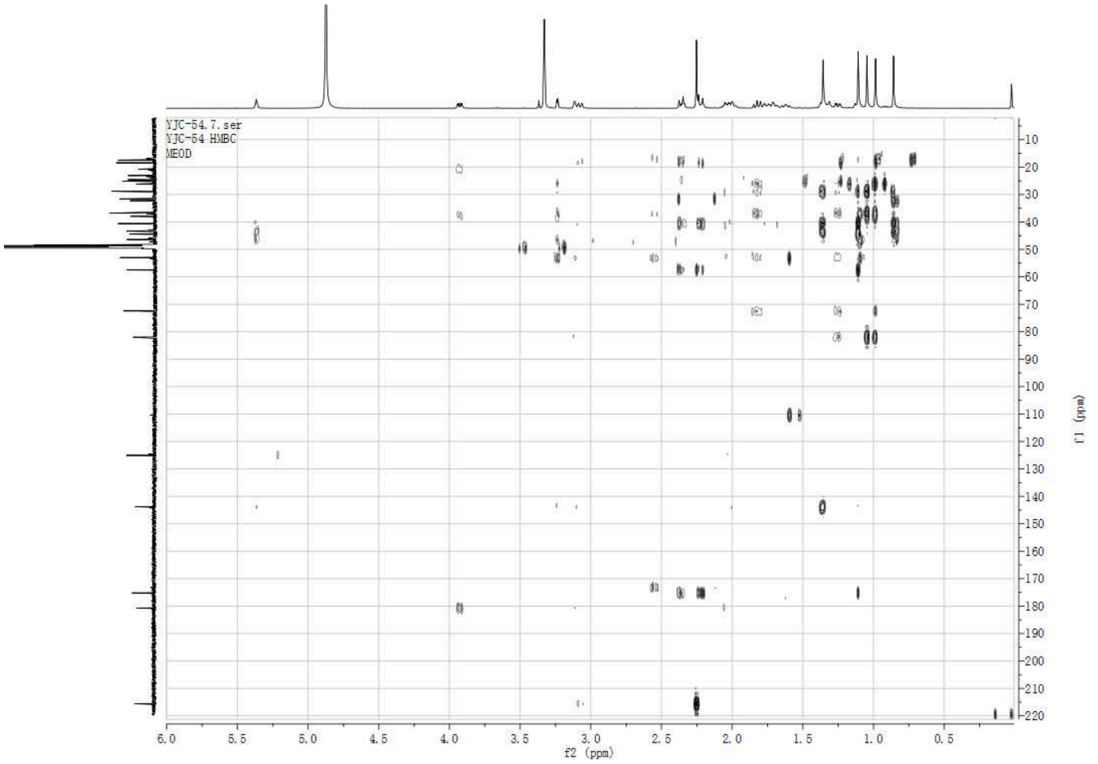

Figure S46. HMBC spectrum of **5**

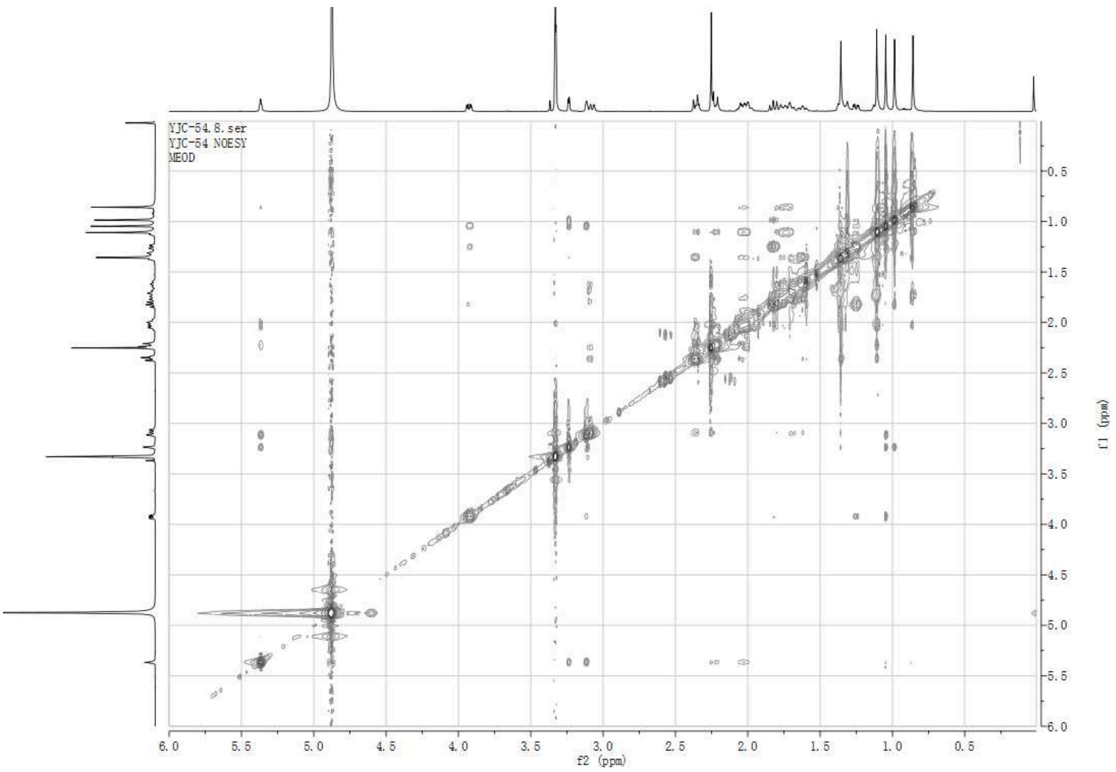

Figure S47. NOESY spectrum of **5**

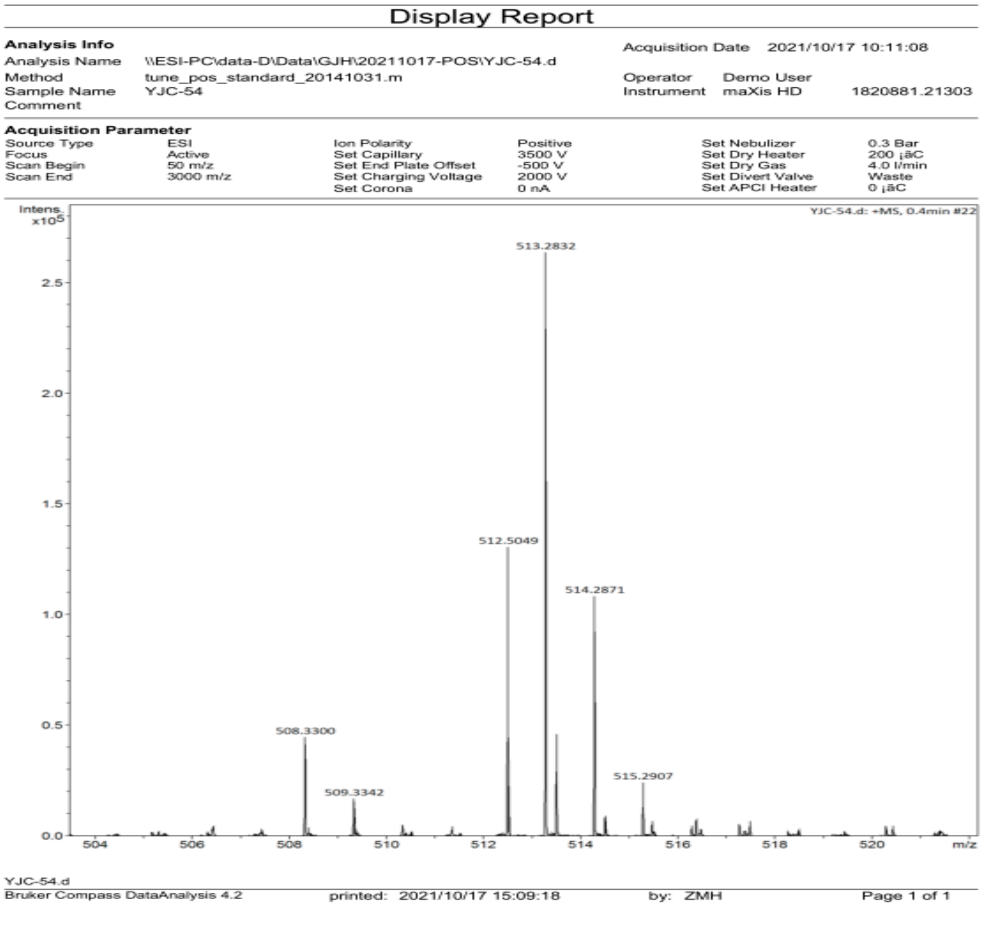

Figure S48. HR-ESI-MS Spectrum of **5**

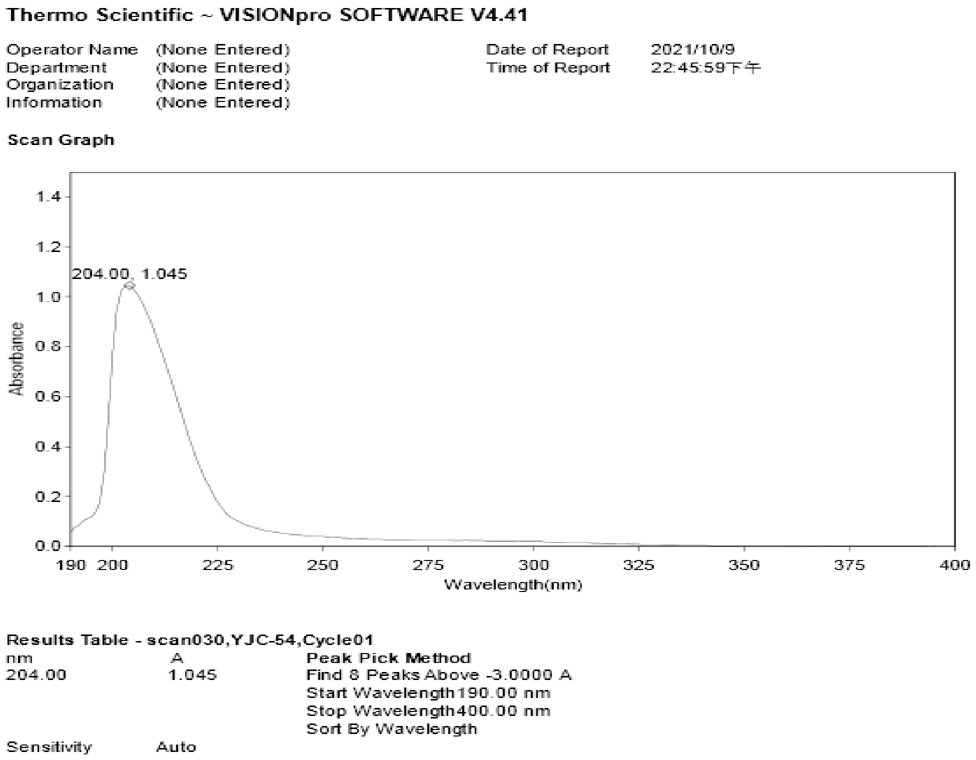

Figure S49. UV spectrum of **5**

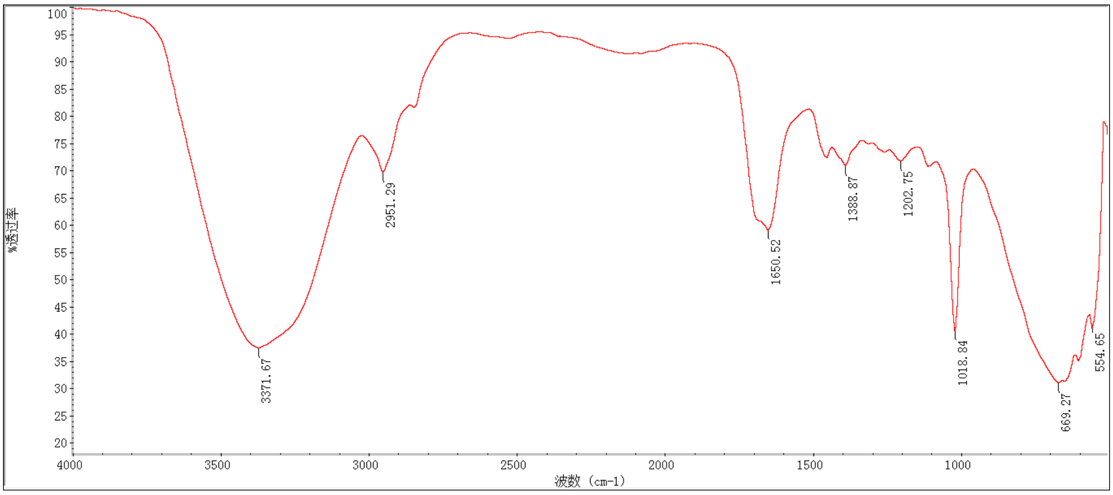

Figure S50. IR spectrum of **5**

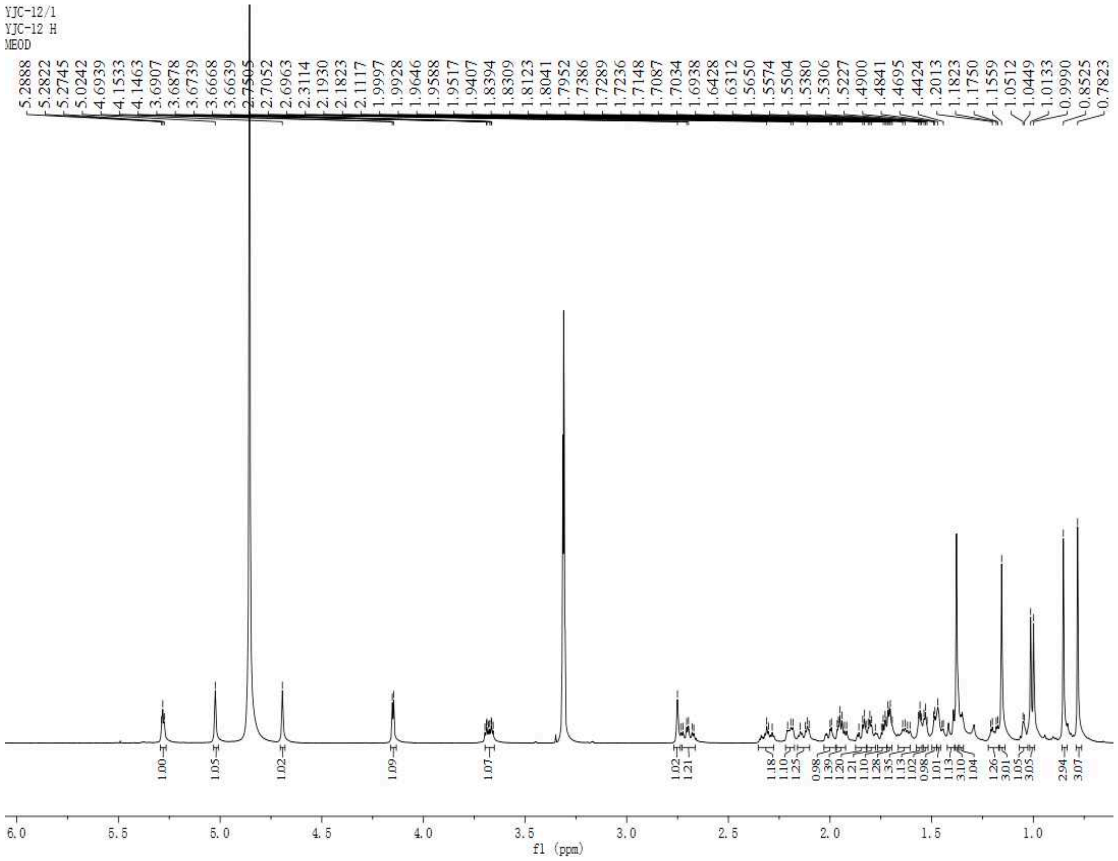

Figure S51. ^{1}H NMR spectrum (500 MHz, CD_3OD) of **6**

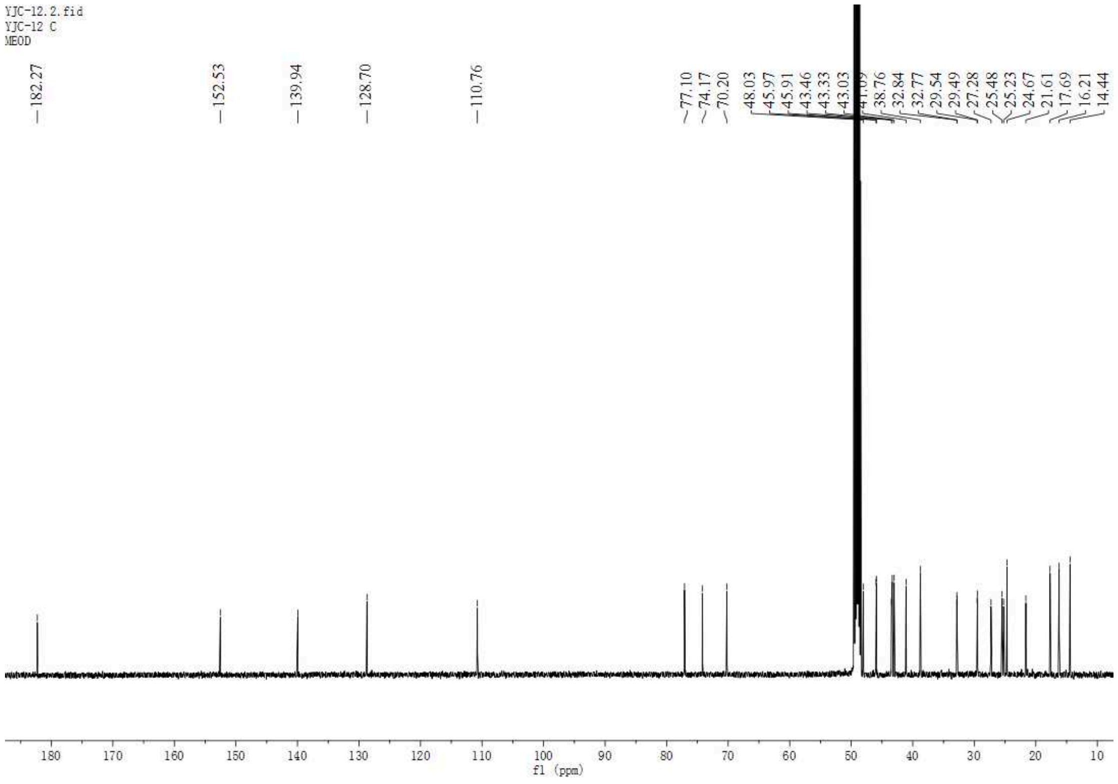

Figure S52. ^{13}C NMR spectrum (125 MHz, CD_3OD) of **6**

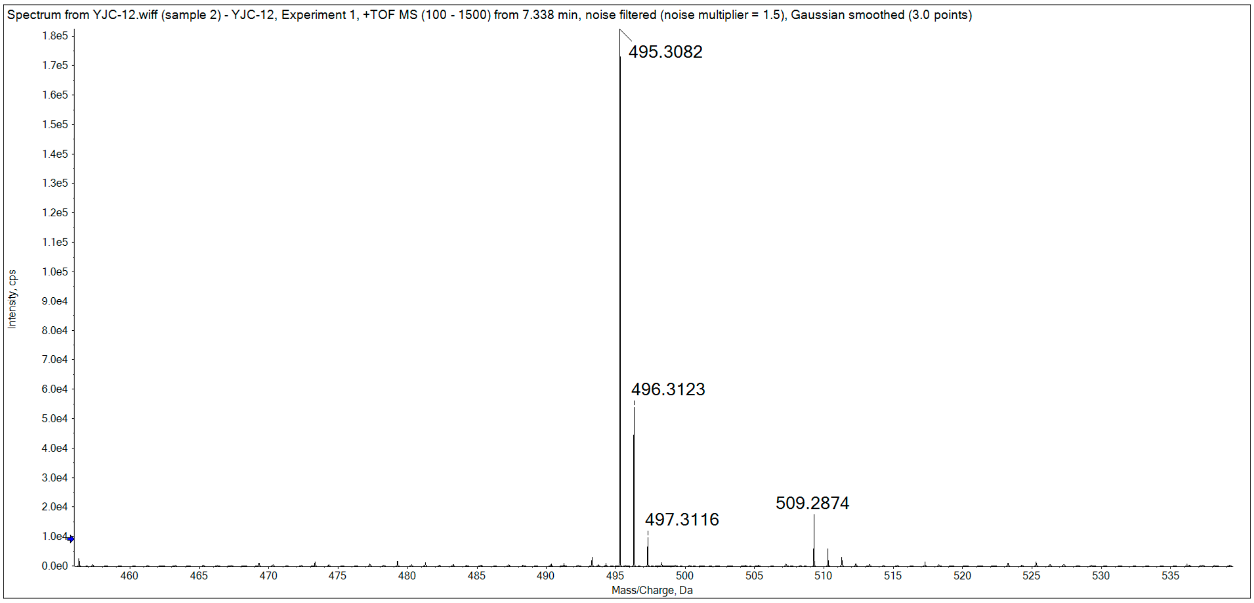

Figure S53. HR-ESI-MS Spectrum of **6**

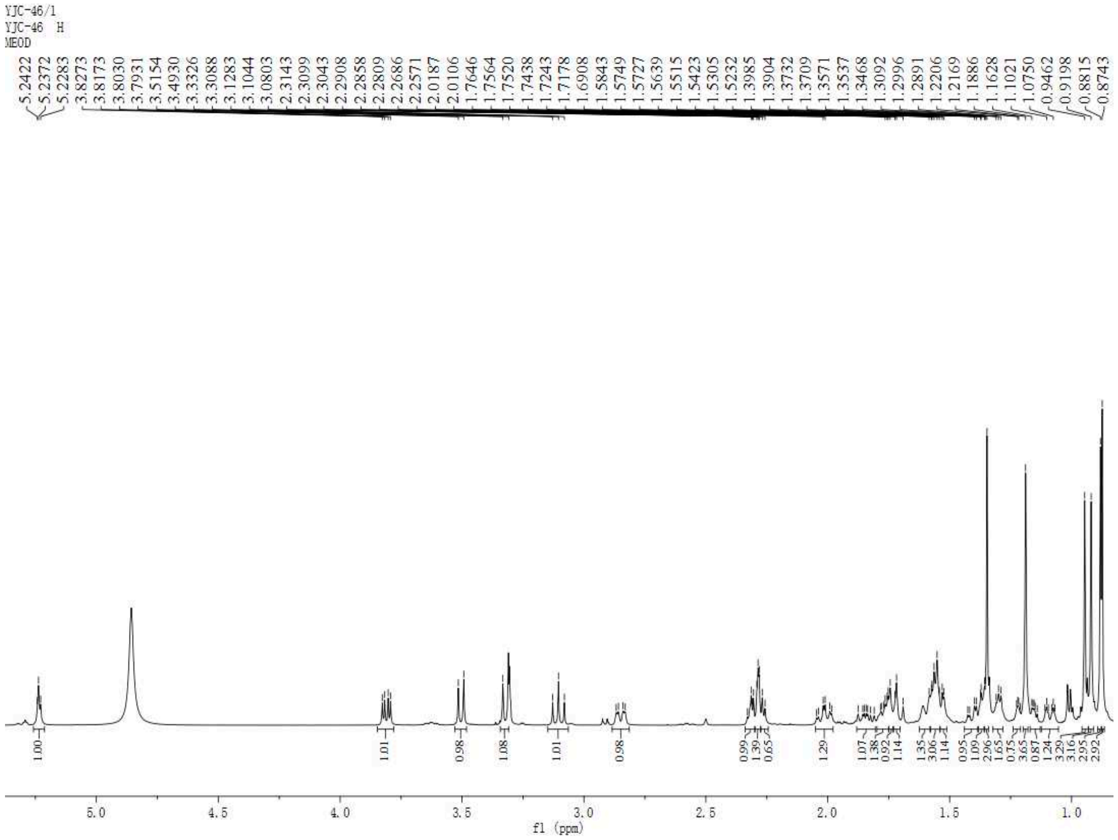

Figure S54. ^{1}H NMR spectrum (500 MHz, CD_3OD) of **7**

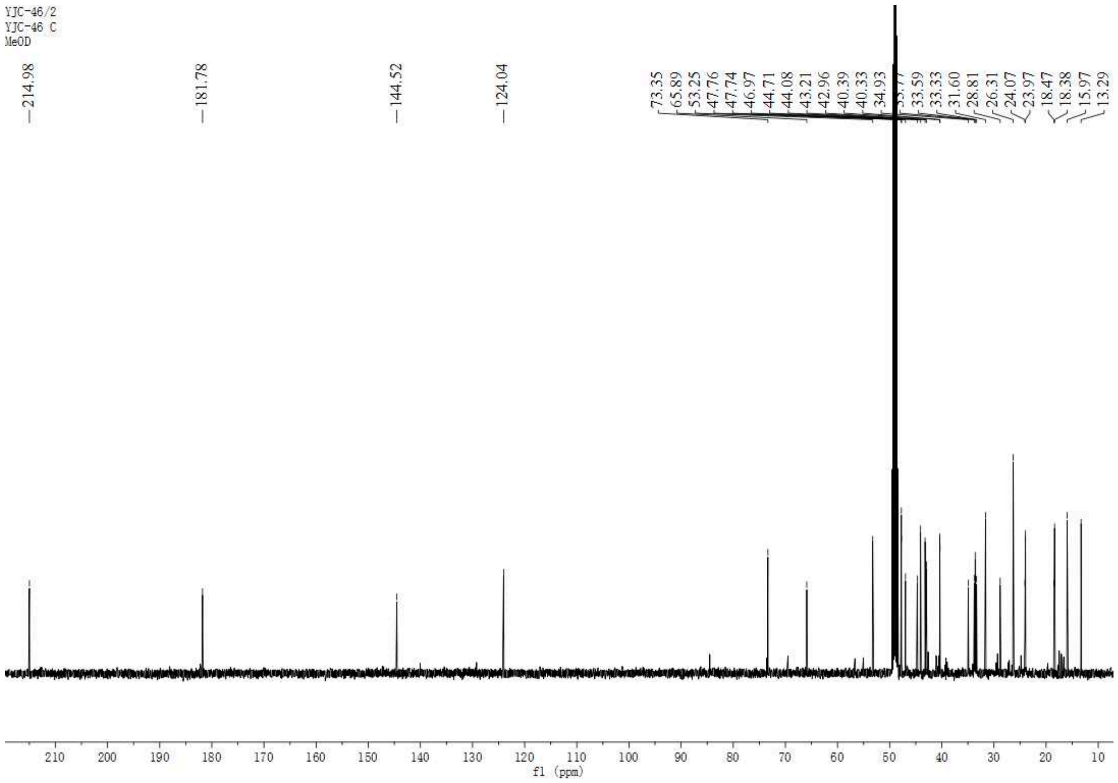

Figure S55. ^{13}C NMR spectrum (125 MHz, CD_3OD) of **7**

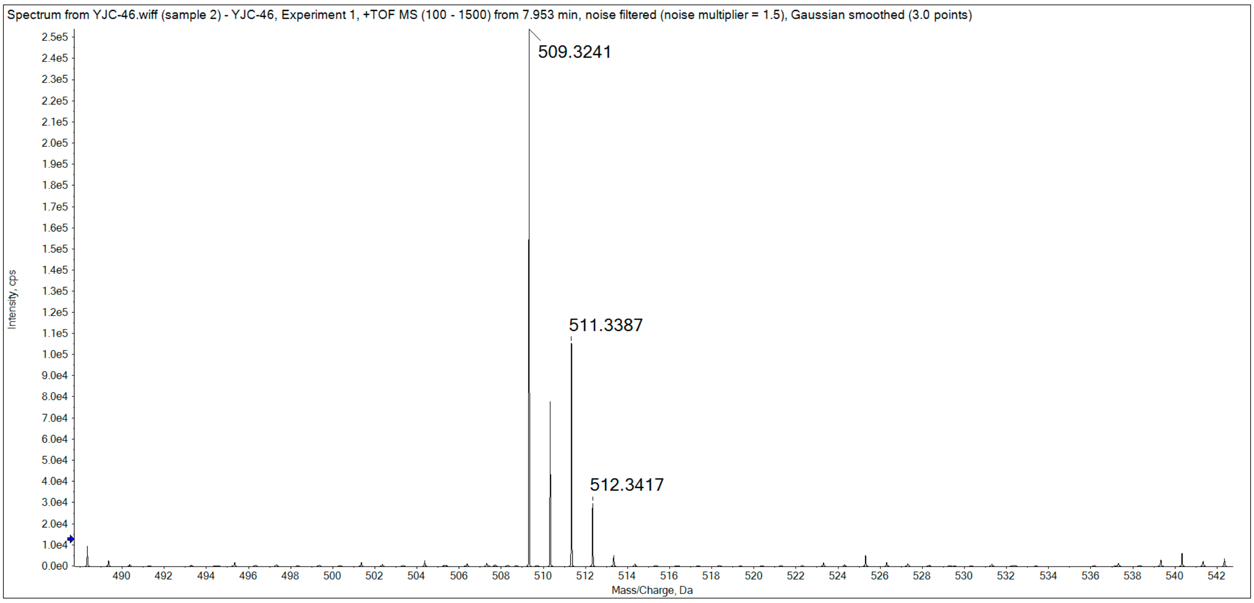

Figure S56. HR-ESI-MS Spectrum of **7**

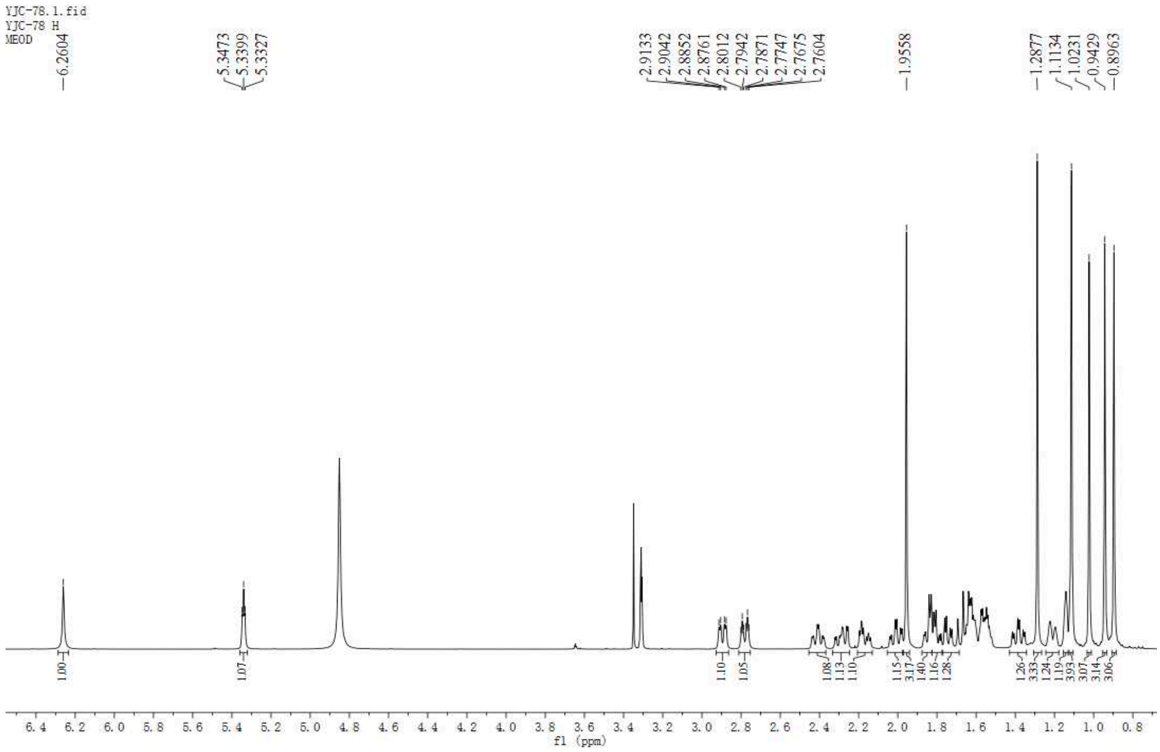

Figure S57. ^{1}H NMR spectrum (500 MHz, CD_3OD) of **8**

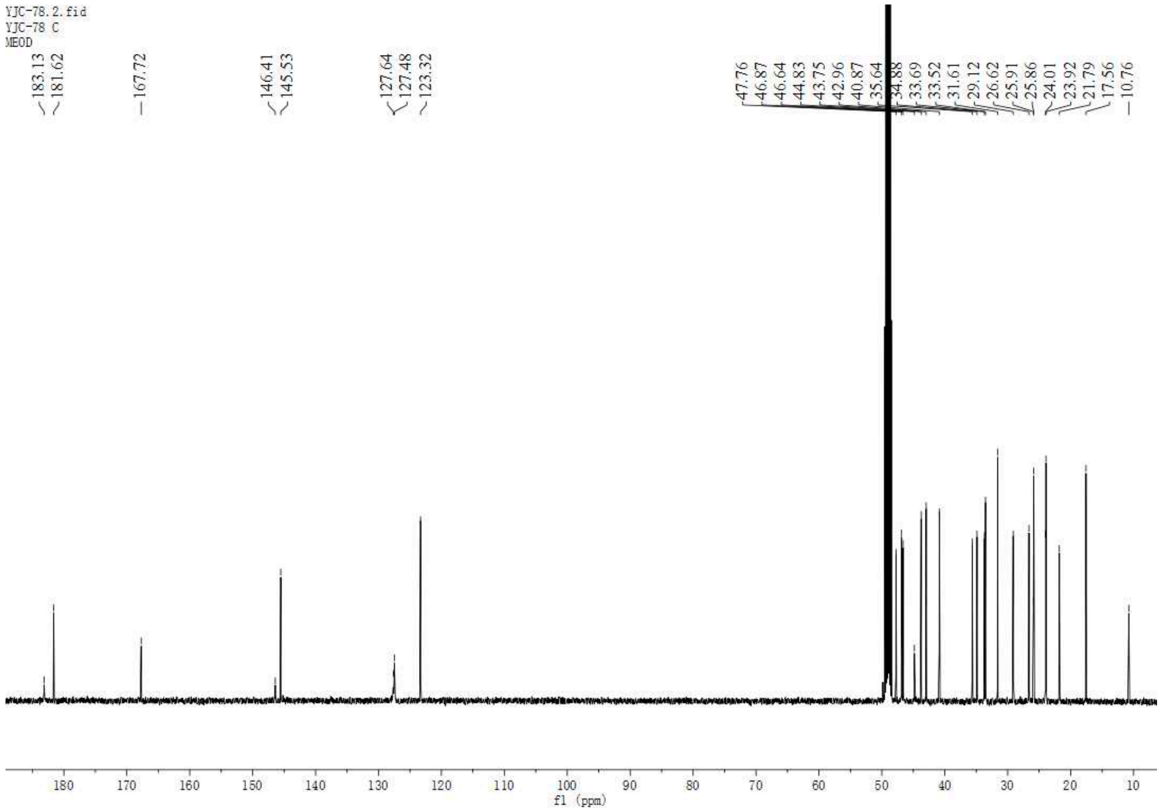

Figure S58. ^{13}C NMR spectrum (125 MHz, CD_3OD) of **8**

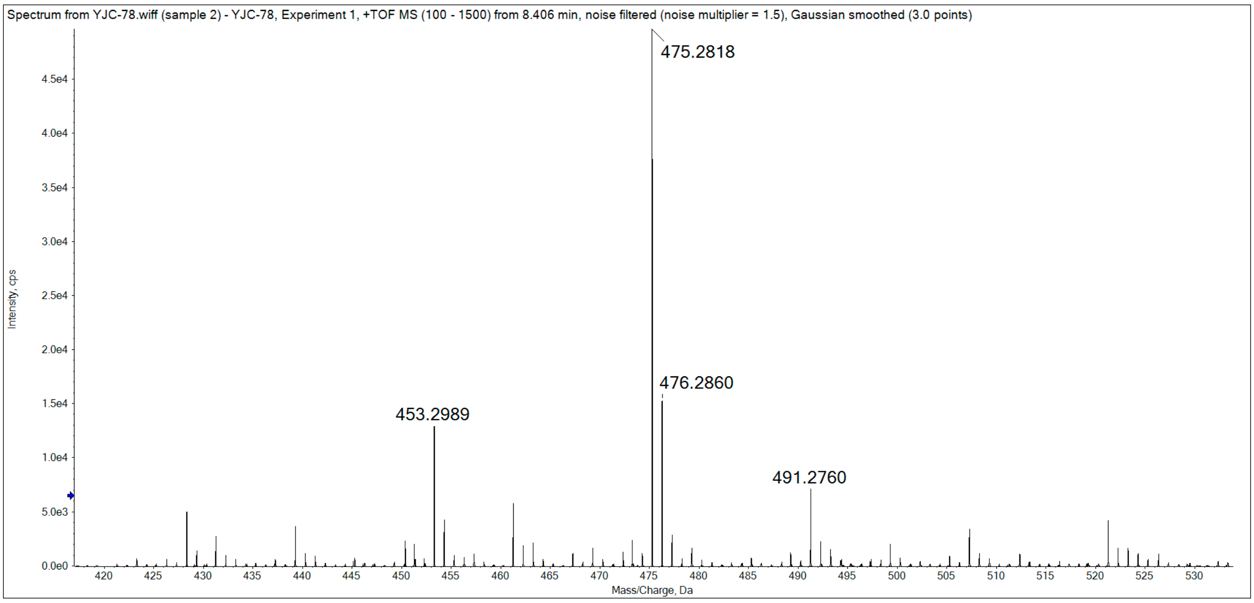

Figure S59. HR-ESI-MS Spectrum of **8**

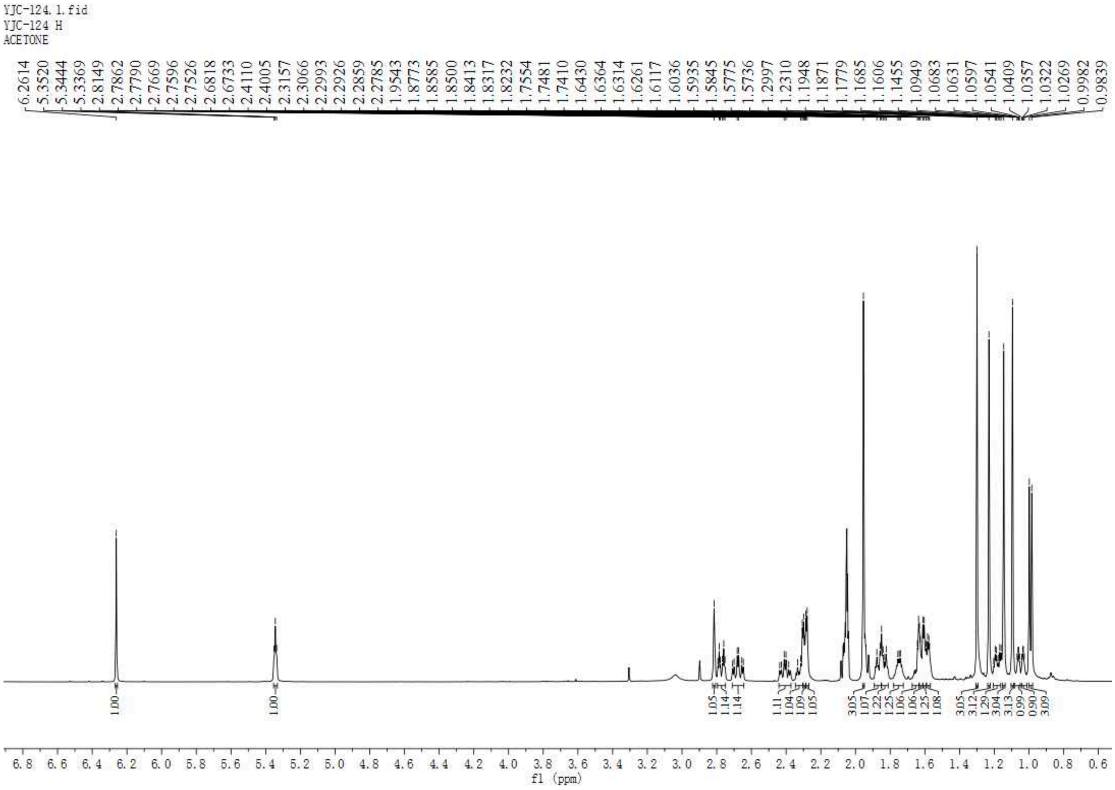

Figure S60. ^{1}H NMR spectrum (500 MHz, Acetone-d_6) of **9**

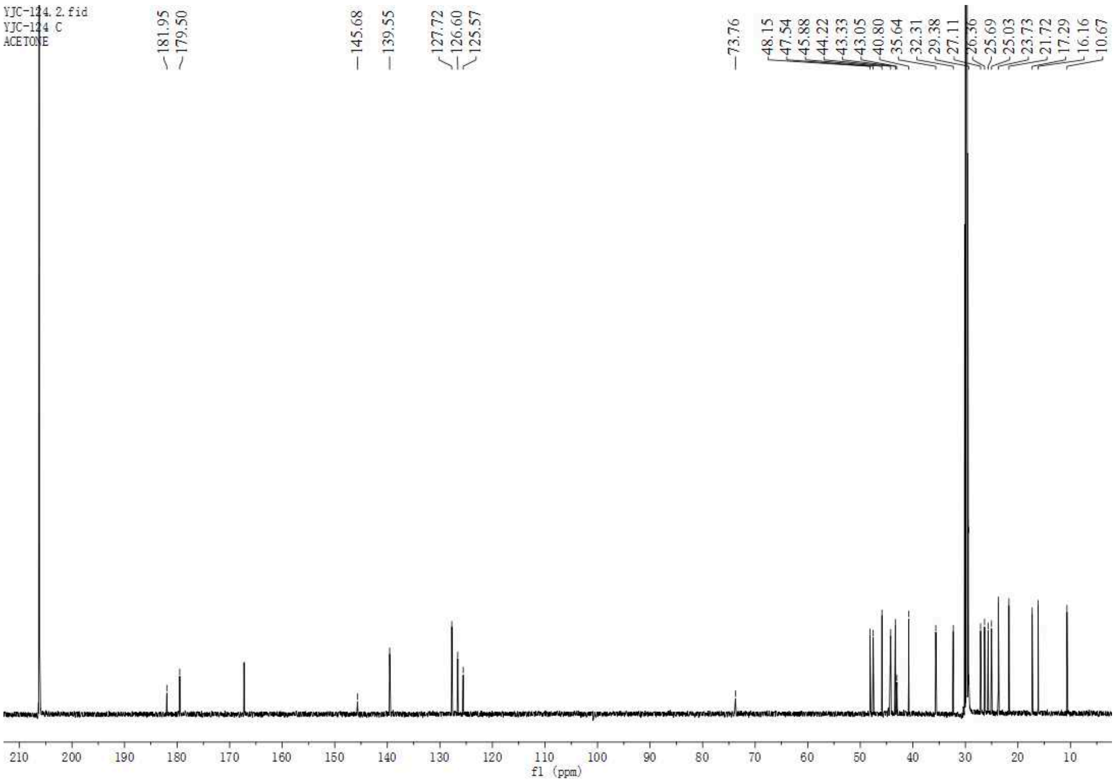

Figure S61. ^{13}C NMR spectrum (125 MHz, Acetone-d_6) of **9**

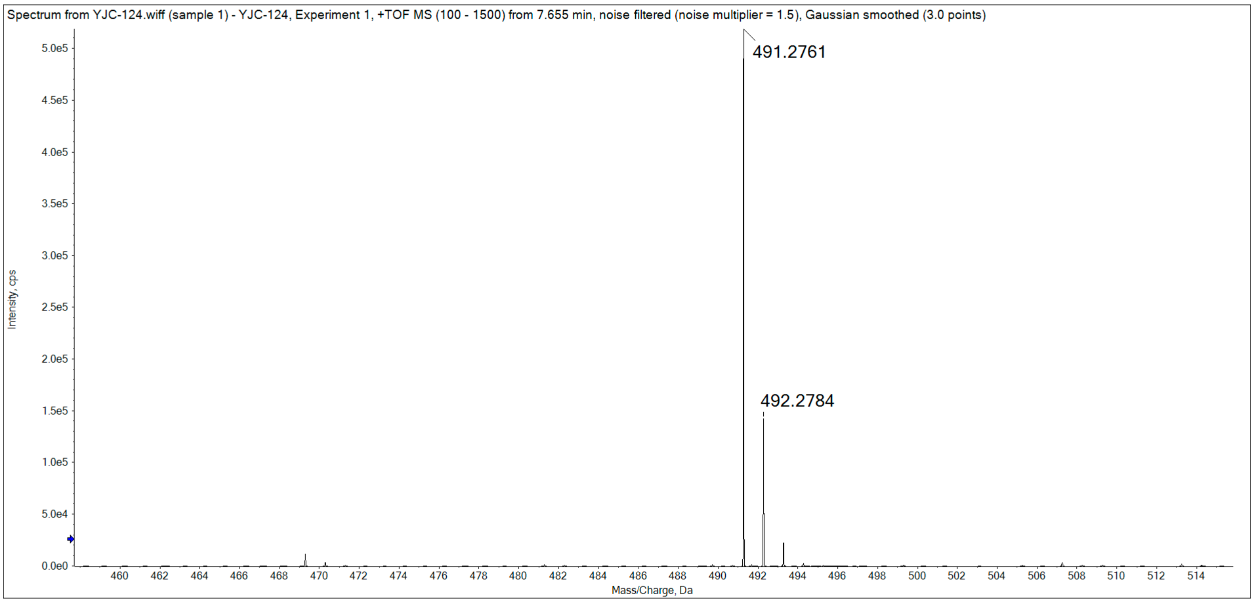

Figure S62. HR-ESI-MS Spectrum of **9**

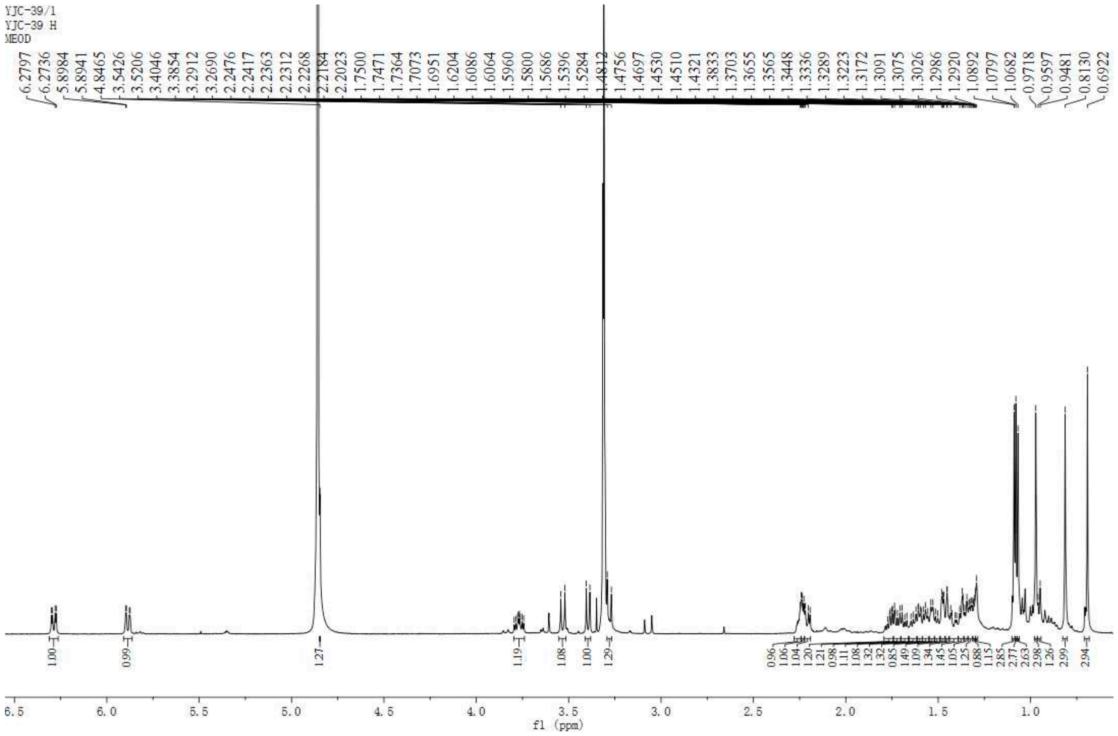

Figure S63. ^{1}H NMR spectrum (500 MHz, CD_3OD) of **10**

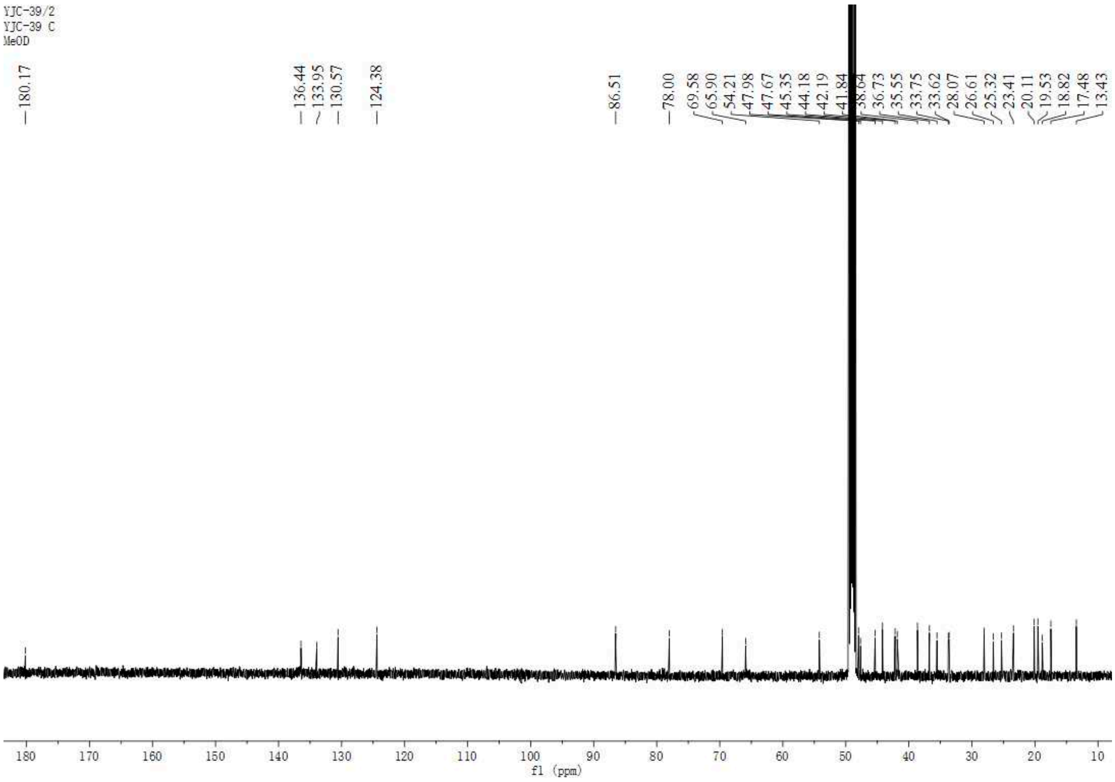

Figure S64. ^{13}C NMR spectrum (125 MHz, CD_3OD) of **10**

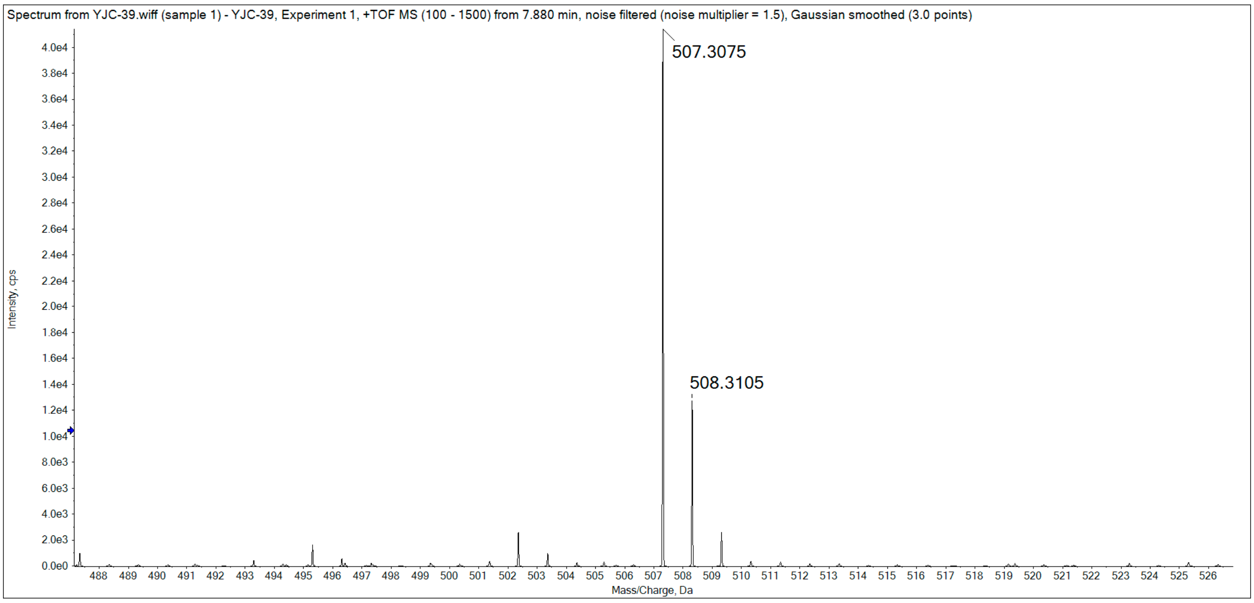

Figure S65. HR-ESI-MS Spectrum of **10**

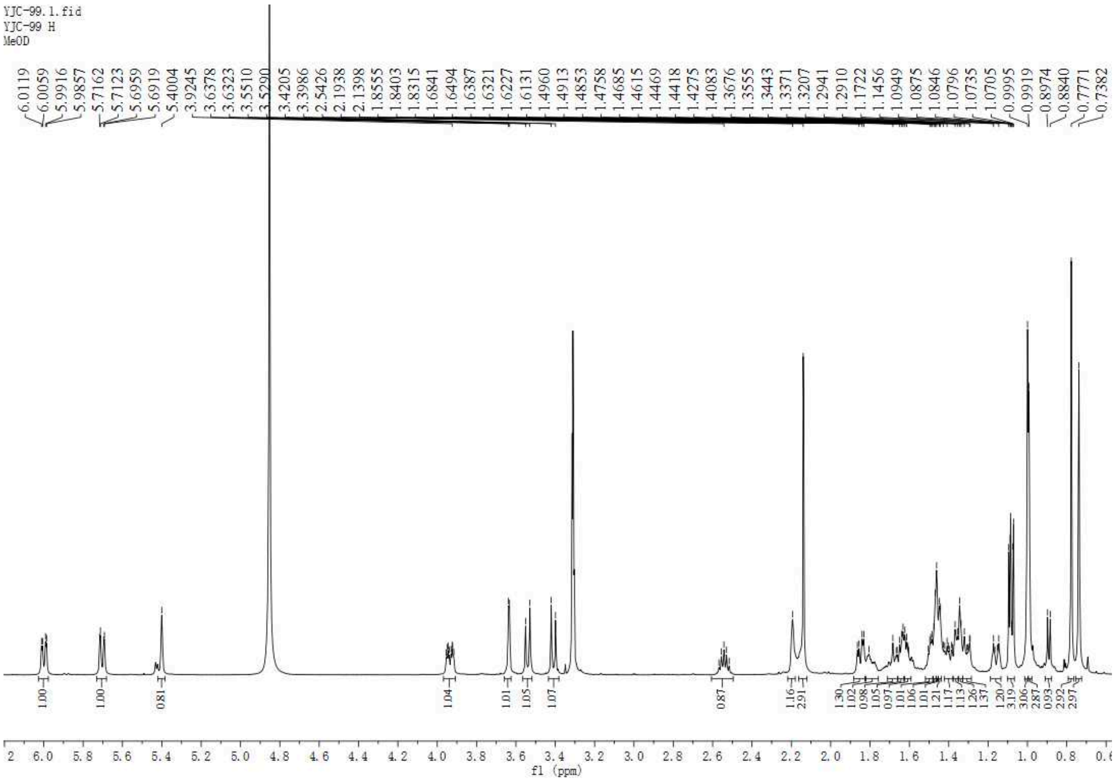

Figure S66. ^{1}H NMR spectrum (500 MHz, CD_3OD) of **11**

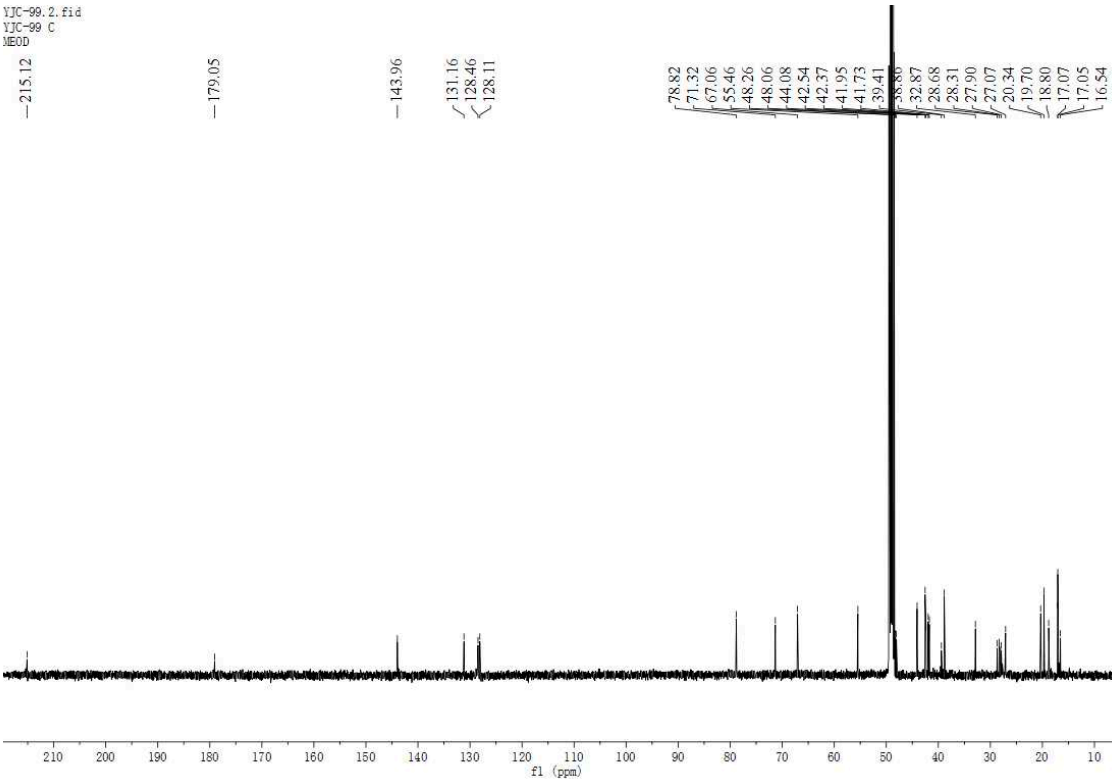

Figure S67. ^{13}C NMR spectrum (125 MHz, CD_3OD) of **11**

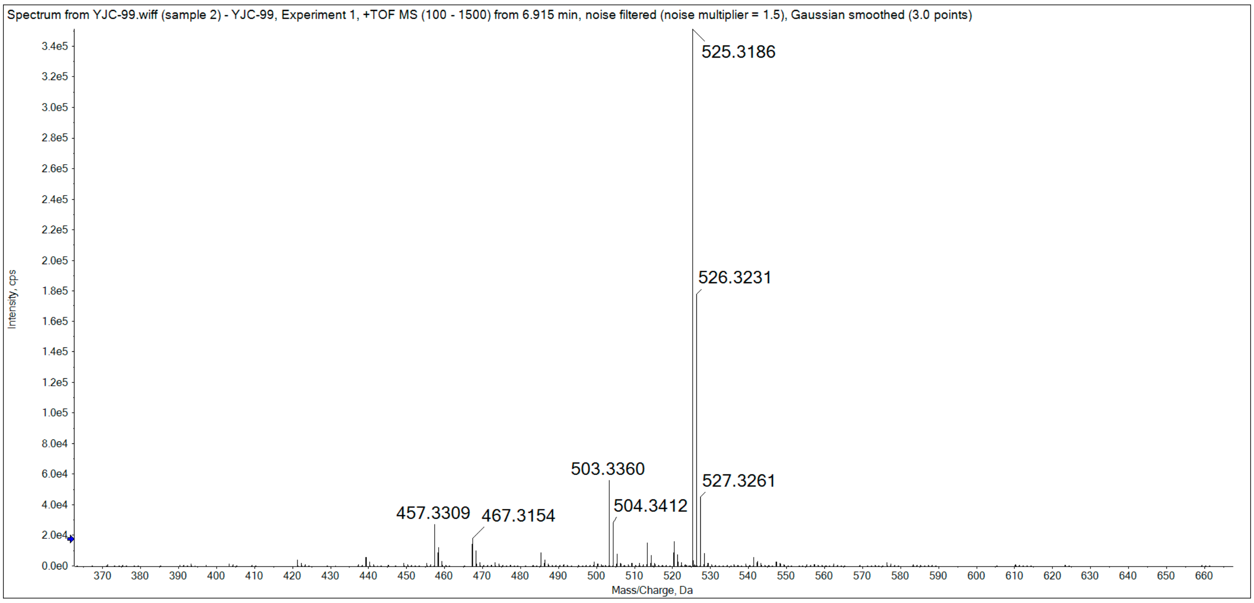

Figure S68. HR-ESI-MS Spectrum of **11**

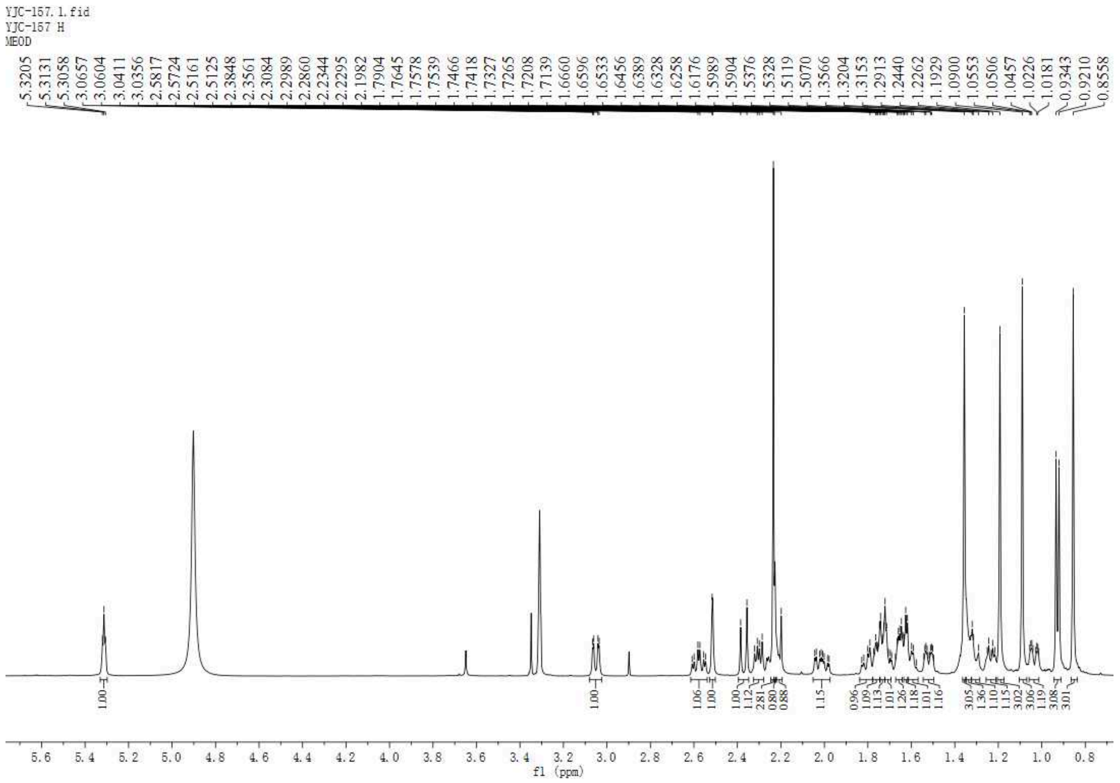

Figure S69. ^{1}H NMR spectrum (500 MHz, CD_3OD) of **12**

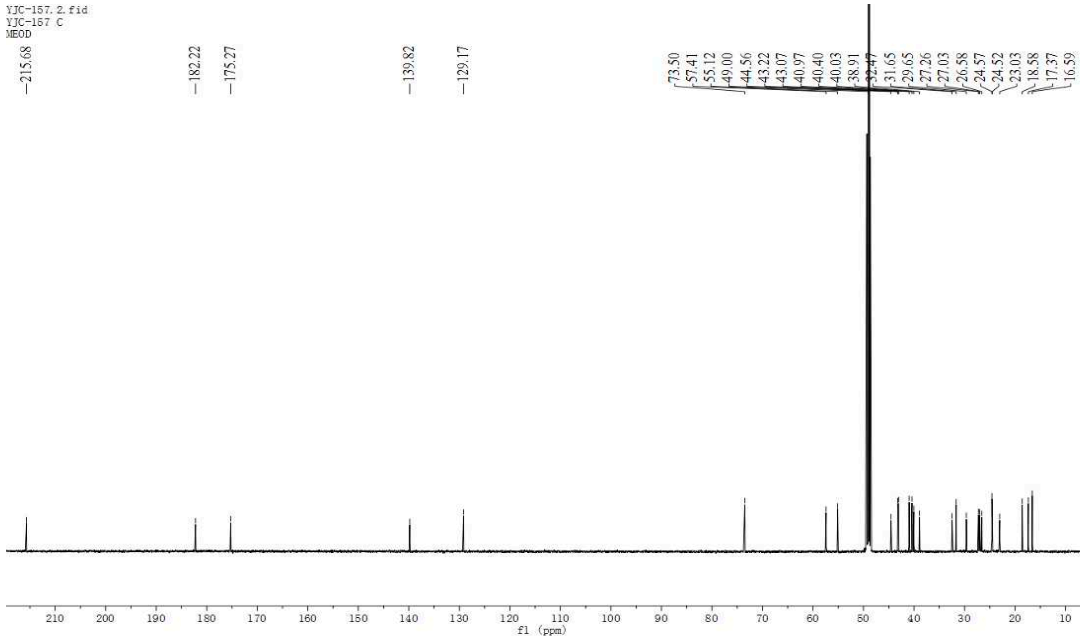

Figure S70. ^{13}C NMR spectrum (125 MHz, CD_3OD) of **12**

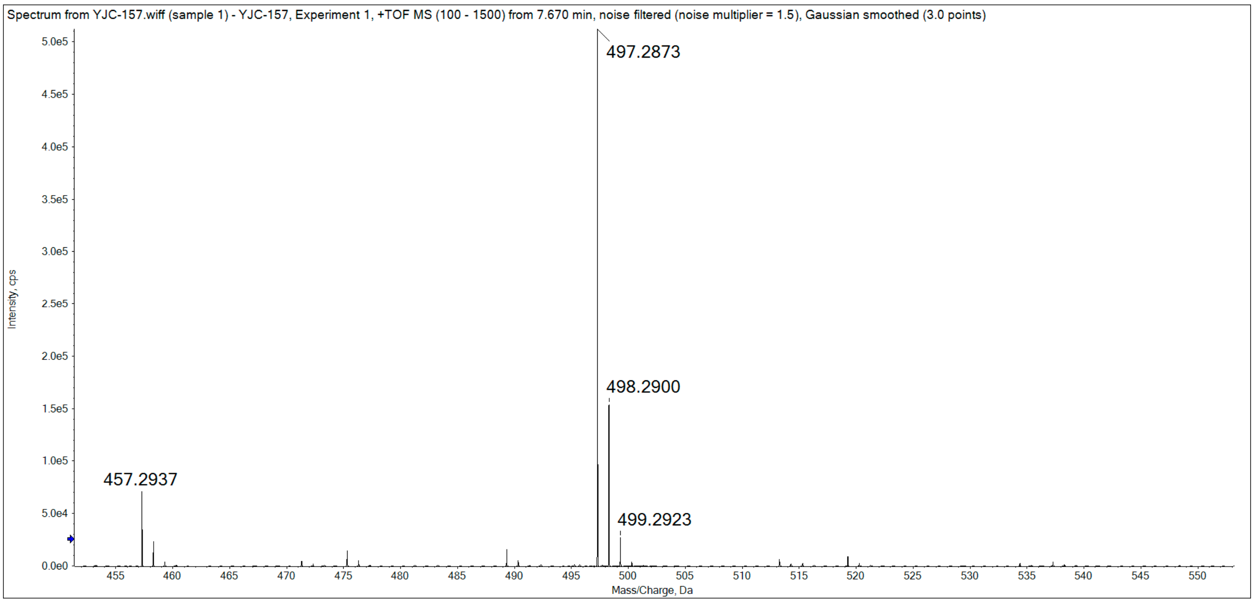

Figure S71. HR-ESI-MS Spectrum of **12**

Table 4 Melting points of compounds **6-12**

compound	mp (℃)	Group	mp (℃)
**6**	234	**10**	253
**7**	245	**11**	240
**8**	215	**12**	238
**9**	241		

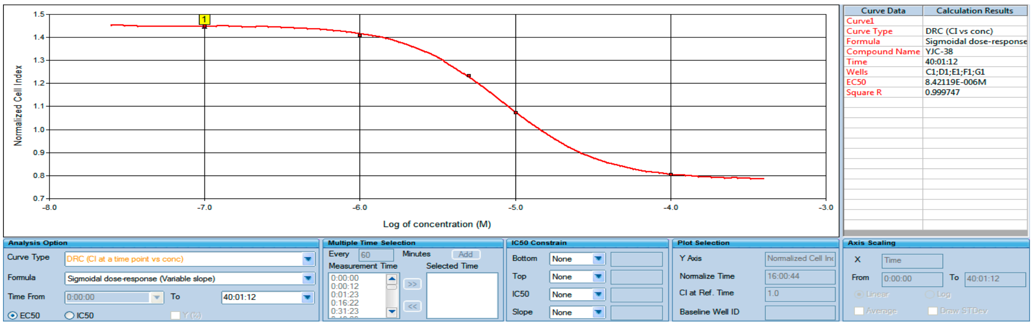

Figure S72. The curve obtained with compound **1**

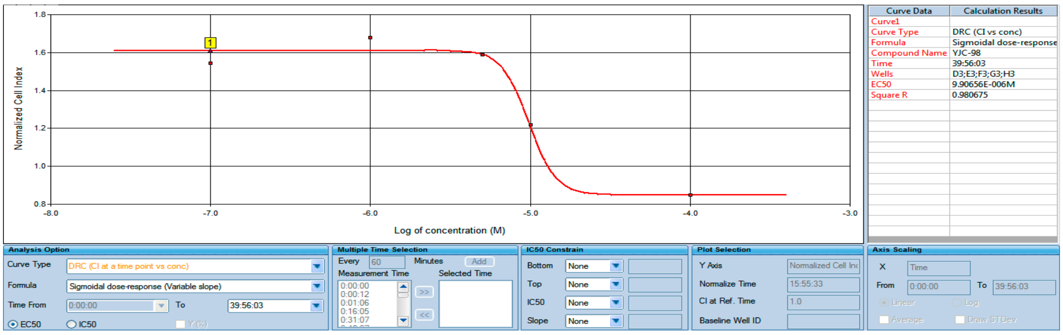

Figure S73. The curve obtained with compound **2**

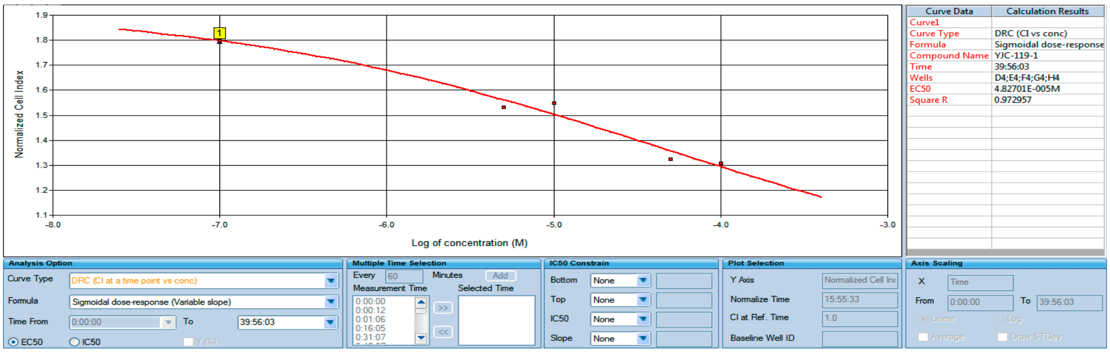

Figure S74. The curve obtained with compound **3**

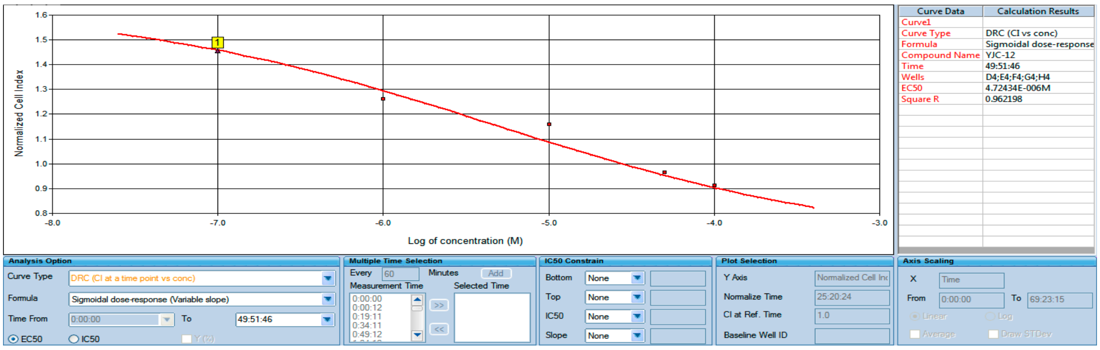

Figure S75. The curve obtained with compound **6**

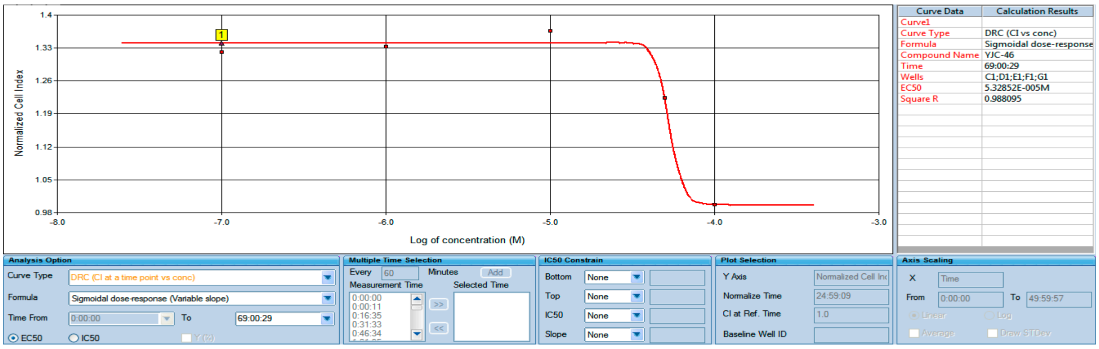

Figure S76. The curve obtained with compound **7**

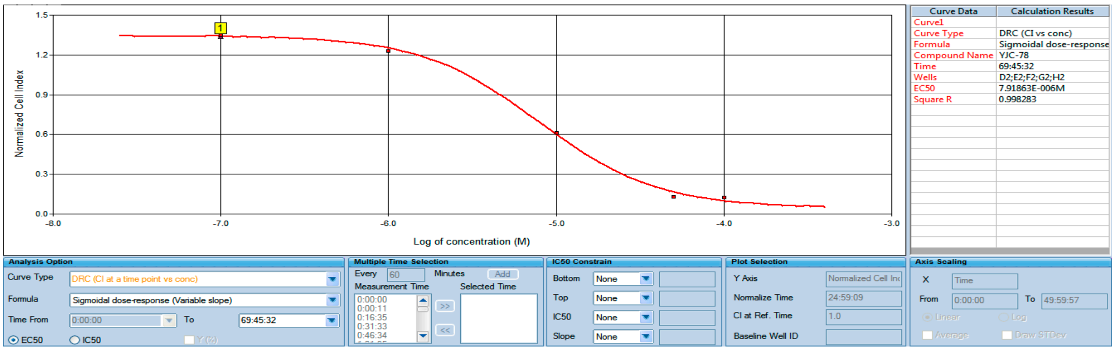

Figure S77. The curve obtained with compound **8**

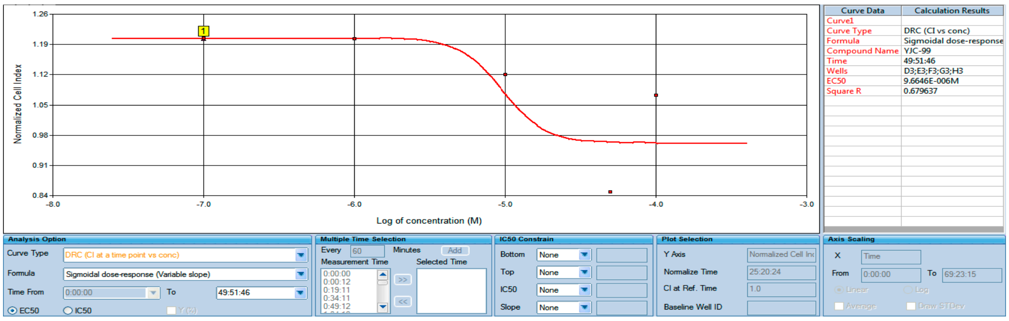

Figure S78. The curve obtained with compound **11**

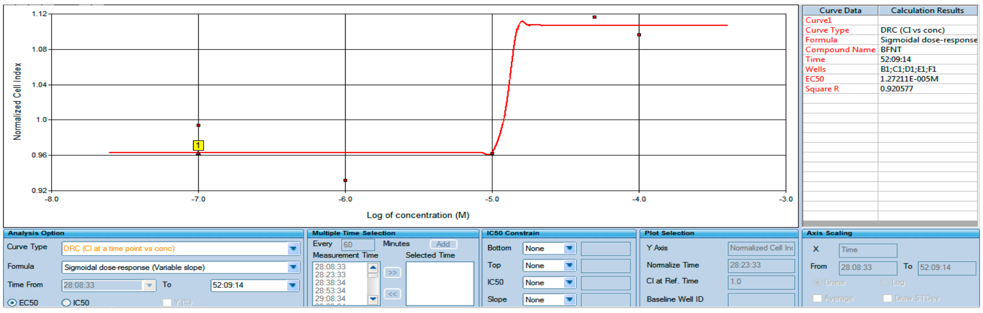

Figure S79. The curve obtained with positive control compound Pirfenidone
